# Canonical cortico-hippocampal dynamics underlie memory of navigational episodes and its early decline in aging

**DOI:** 10.1162/IMAG.a.101

**Published:** 2025-08-18

**Authors:** Jaeseob Lim, Sang-Eon Park, Sang-Hun Lee, Sang Ah Lee

**Affiliations:** Department of Brain and Cognitive Sciences, Seoul National University, Gwanak-gu, Seoul, Republic of Korea

**Keywords:** spatial memory, navigation, BOLD dynamics, individual to canonical similarity, intersubject functional connectivity, aging, hippocampus, medial temporal lobe

## Abstract

Successful encoding of a navigational episode entails the dynamic processing of perceptual information, time-locked to the appearance of salient landmarks and turns along the way. We hypothesized that identical navigational experiences will be represented in a similar manner across individuals and that a deviation from such canonical dynamics in the cortico-hippocampal network may underlie differences in navigational memory across individuals and its decline in aging. 76 participants (42 females) across two age groups (young: 20–30 years, aging: 50–65 years) watched 24 different 1-minute-long first-person-view virtual navigation videos in the fMRI scanner, followed by a memory question about the traveled path or destination. Canonical dynamics were defined as the averaged neural dynamics across participants during the navigation period for each brain region. First, we found that individual-to-canonical similarity (ICS) was highest in the dorsal/ventral visual streams and precuneus and was linked to informative navigational events, such as turning or landmark viewing. ICS in the medial temporal lobe (MTL) and the coordinated activity between the hippocampus and canonical cortical dynamics were strongly associated with individual spatial memory performance. Moreover, a reduction in the intersubject functional connectivity between the MTL and the canonical cortical dynamics mediated the effects of aging on cognitive performance, highlighting its role in navigation and episodic memory. Complementary to traditional time-averaged activation measures, canonical neural dynamics may be particularly revealing of how the brain processes information across spatiotemporally extended events.

## 1 Introduction

Spatial navigation is a highly complex process involving sequential processing of spatial information such as turns and landmarks along an extended path. For effective encoding of navigational episodes, neural responses should dynamically change in response to moment-to-moment variations in spatial informational content and be reflected in their transformation into memory representations (Ekstrom & Hill, 2023; R. A. Epstein et al., 2017). An investigation of dynamic neural activity may be particularly effective in understanding the neural underpinnings of navigation and may manifest in a canonical manner across individuals that experience the same navigational episode.

Univariate analyses that average neural activation across time may not be fit for capturing dynamic activities for naturalistic and temporally-extended complex stimuli (Ben-Yakov et al., 2012; Hasson et al., 2010). As an alternative, measuring activation dynamics shared across participants (Hasson et al., 2004; Nastase et al., 2019) provides a complementary method of identifying neural correlates of cognition by characterizing reliable neural responses across time. This approach can identify brain regions associated with task conditions and performance that are not detectable using conventional methods applied to the same dataset (Hasson et al., 2010; Jangraw et al., 2023; Wilson et al., 2008).

Due to its ease of application to naturalistic stimuli without requiring a specific model, the inter-subject correlation method has been widely used during movie watching (Campbell et al., 2015; Hasson et al., 2008; Ki et al., 2016), social interactions (Hasson et al., 2012), and narrative comprehension (Baldassano et al., 2017; Jangraw et al., 2023; Simony et al., 2016; Wilson et al., 2008), revealing high neural synchronization across individuals experiencing the same event (Hasson et al., 2004, 2010). Based on these previous studies, we hypothesized that when individuals are presented with identical spatiotemporally dynamic navigational episodes, there should be a large degree of similarity in the neural processing of information across events. These “canonical” neural dynamics shared across individuals during navigation may reveal distinct brain activities that correspond to the processing of important spatial events such as landmarks and turns (Indovina et al., 2016; Janzen & Van Turennout, 2004; Javadi et al., 2019).

Our hypothesis makes several predictions: First, canonical dynamics during navigation should be observed in brain regions related to spatial perception and memory. Second, the dynamic neural activity should be time-locked to informative spatial events, making each navigational episode decodable from its canonical dynamics (Chen et al., 2015; Haxby et al., 2011). Furthermore, participants whose neural activities show high correspondence with the canonical dynamics should perform better (Chamberlain et al., 2024; Hasson et al., 2008; Jangraw et al., 2023; Sheng et al., 2023) in spatial memory than those who do not. Not only that, based on the significance of medial temporal lobe (MTL) function to navigation, the alignment in neural dynamics between the hippocampus and cortical input regions should be highly indicative of spatial memory performance. Finally, over the course of aging, as hippocampal function declines, the MTL should deviate from the canonical cortical activity and lead to lower performance in older adults.

**Fig. 1.**
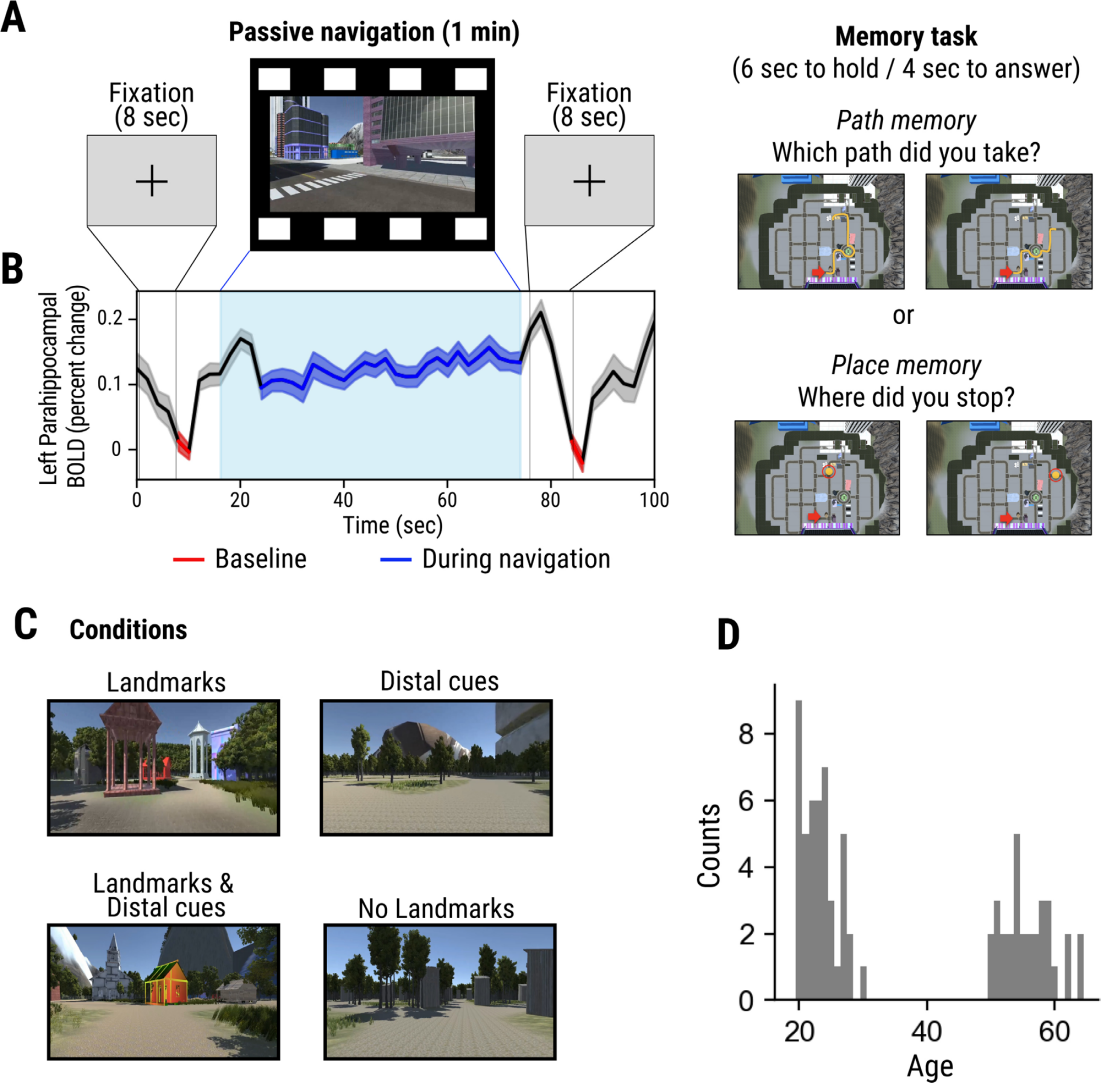
Experiment and task design. (A) Each trial began with a fixation period, followed by a top-down-view map (see Section 2). Participants watched a 1-minute video of a spatial navigation episode. Afterward, a second fixation and map presentation followed. Finally, participants performed a memory test on the navigation content. (B) An example of a BOLD fMRI time series across the whole task period. (C) Four different conditions varying in the presence of landmarks and distal cues. Each condition consisted of 6 different episodes in 4 different environmental contexts for a total of 24 trials. (D) Distribution of participants’ age.

To test the above predictions, we collected fMRI data from a total of 76 participants across two age groups (young: 20–30 years; aging: 50–65 years). Participants watched 24 one-minute-long videos of navigational episodes and subsequently answered spatial memory questions about each episode (Fig. 1A, B). The videos varied in their turn sequences and availability of landmark cues (Fig. 1C, four different conditions: landmarks, distal cues, landmarks & distal cues, and no landmarks) and consisted of four different environments built using Unity software (Supplementary Fig. 1). We used averaged blood-oxygenation-level-dependent (BOLD) time series data to identify the brain regions showing shared neural dynamic activities across individuals during navigation and to characterize whether spatially informative components (e.g., turns and landmarks) evoke specific neural activities across brain regions. We then compared the performance between those with low and high individual-to-canonical similarity (ICS). We identified the relevance of each brain region’s dynamic activity to spatial memory by performing correlations with task performance and further explored the functional significance of corresponding dynamics between the hippocampus and cortical regions. Lastly, we tested whether a deviation from the canonical neural dynamics can be observed in aging participants (Campbell et al., 2015; Geerligs & Campbell, 2018; Petroni et al., 2018) and explored its potential effects on age-related decline by conducting a mediation analysis across age, degree of similarity to the canonical dynamics, and spatial memory performance.

## 2 Methods

### 2.1 Participants

76 healthy adults (42 females, aged between 20 and 65, mean = 36.54, SD = 16.36) were recruited from the local community. Prior research has demonstrated that cognitive decline and memory deficits begin in middle age (Ballesteros et al., 2009; Dohm-Hansen et al., 2024; Statsenko et al., 2021); based on our previous EEG study using the same paradigm that found a significant age-dependent decrease in task performance among participants aged 22 to 63, we recruited participants within a similar age range. The younger group consisted of 45 participants aged 20–30 (25 females; mean = 23.22, SD = 2.63), and the older group consisted of 31 participants aged 50–65 (17 females; mean = 55.87, SD = 3.92). All participants provided informed consent, and the study was approved by the Institutional Review Board of Seoul National University. All participants had normal or corrected-to-normal vision and reported no history of neurological or psychiatric disorders. Participants were paid 40,000 KRW for completing the entire experiment.

### 2.2 Stimuli

We created 24 videos of 1-minute-long passive first-person-view navigation episodes in virtual environments using Unity (https://unity.com/). The passive paradigm was adopted to induce participants to process spatial information (Marchette et al., 2011) while making the timing of all encoding episodes identical across participants. Each video was unique in the combination of path sequence (consisting of 4-8 turns), presence of landmarks (unique buildings and distal cues), and overall environmental context (village forest, desert, and city). Participants observed the stimuli by looking through an inclined mirror connected to the head coil inside the MR scanner, enabling visualization of the stimuli projected via an LCD projector onto a screen positioned at the terminus of the magnet bore, with a viewing distance of 87 cm, resulting in a field of view of 22 x 17°. The behavioral task was programmed using MATLAB (MathWorks) and Psychtoolbox (http://psychtoolbox.org/). We defined three types of spatial cues: First, uninformative buildings, which had similar shape, size, and color (e.g., container boxes). These buildings were difficult to distinguish from each other and, therefore, were hard to associate with specific locations. As a result, they play a limited role as landmarks during navigation. Six of the episodes (“No Landmark” condition) included only uninformative buildings. Second, unique buildings and objects that were easily associated with specific locations on the map were defined as local landmarks. Six episodes included this type of landmark. Lastly, large distal structures, such as mountains, functioned as directional cues in navigation (rather than being associated with specific locations). We refer to these structures as distal cues, and six episodes included only this type of structure. These landmark conditions were designed based on past studies in both human and nonhuman animals which showed that local environmental landmarks and distal orientation cues are involved in distinctive navigational processes (e.g., local landmarks for the recognition of spatial location and distal landmarks for the computation of heading and orientation) (Chan et al., 2012; Knierim & Hamilton, 2011; Lester et al., 2017). The remaining six episodes included both distal cues and local landmarks (Fig. 1C). Here, we will use the term ‘landmark’ to refer specifically to the second type (local landmarks), distinguishing them from distal cues or uninformative buildings.

### 2.3 Behavioral task

Participants were provided with detailed instructions on the task before entering the fMRI scanner. The experiment consisted of 4 runs, each containing 6 trials; participants were able to take a rest between runs if needed. Each trial began with an 8-second fixation on a cross at the center of the screen, followed by an 8-second presentation of an overhead-view map of the virtual environment, marked with a red arrow indicating the starting point and the initial heading direction. Then, participants watched a navigational episode for 1 minute. The virtual navigator was stationary during the first and last 2 seconds of the video and moved at a consistent speed during the rest of the period. After the video ended, a fixation cross appeared for 8 seconds followed by the reappearance of the overhead-view map for 4 seconds, during which participants were instructed to recall the navigational episode while viewing the map. Then, participants were presented with two overhead-view maps that either marked the destination (place) or the path and asked to choose the correct one (Fig. 1A, a yellow line marked the trajectory for path memory questions, and a yellow circle marked the targets for place memory questions). Participants solved a total of 12 path and 12 place memory questions and selected the correct option by pressing the left or right button. Participants were not allowed to answer in the first 6 seconds of the question phase to secure enough time without motion-related signals in the data analyses. After 6 seconds, the boundary color of the maps changed to white, at which point participants were given 4 seconds to enter their responses using a button box. The inter-trial interval was adjusted on each trial to fix the length of each trial to 102 seconds.

### 2.4 MRI data collection and preprocessing

MRI data were acquired using a 3T Siemens MAGNETOM Trio scanner equipped with a 32-channel head coil at the Department of Brain and Cognitive Sciences at Seoul National University. T1-weighted MPRAGE structural images were acquired before the beginning of the behavioral task with the following parameters: matrix size = 256 × 256; 172 sagittal slices parallel to the AC-PC axis; voxel size = 1 × 1 × 1 mm^3^; FoV = 256 × 256 mm^2^; TR = 1800 ms; TE = 2.52 ms; flip angle = 9°. Functional data were collected by gradient echo-planar imaging (EPI) functional scans with the following parameters: 44 transversal slices parallel to the AC-PC axis; 3 mm slice thickness; 10% interslice gap; matrix size = 64 × 64; voxel size = 3 × 3 × 3.3 mm^3^; FoV = 192 × 192 mm^2^; TR = 2000 ms; TE = 20 ms; flip angle = 82°. The scanning was stopped and restarted every run (i.e., 6 trials). We used a relatively short echo time to minimize susceptibility-related signal dropout in the medial temporal lobe, a region crucial for navigation studies.

The preprocessing of fMRI data was conducted by using fMRIPrep version 23.1.3 (Esteban et al., 2019) and included slice-timing correction, head motion correction, and coregistration to the T1 structural image. We averaged the preprocessed BOLD signal across voxels within a specific region at each timepoint to estimate a time series of neural activities. These activities were then transformed into a percent signal change time series using the formula: $y\;=\;\frac{x-\bar{x}}{\bar{x}}\;\times \;100$ , where x and $\bar{x}$ represented the preprocessed BOLD signal and its mean over one run, respectively. We then regressed out confounding factors from the percent signal change: confounding variables included 24 motion parameters (comprising 3-axis rotations, 3-axis translations, their temporal derivatives, squared terms, and squared derivatives), framewise displacement, and 5 discrete cosine transform basis functions to address motion-related artifacts and slow drifts in the BOLD signal. Then, the cleaned percent change time series was z-scored to balance individual contributions to the canonical dynamics to avoid having participants with a higher amplitude of percent signal changes disproportionately influence the subsequent results. All steps of signal processing described here—transformation into percent signal changes, regressing out confounding variables, and z-scoring—were performed on a run-by-run (i.e., every 6 trials) basis.

### 2.5 Definition of cortical regions

Segmentation of cortical regions was based on the Human Connectome Project Multimodal Parcellation (Glasser et al., 2016) and resulted in 360 small-scale local regions (180 for each hemisphere), categorized into 22 larger-scale bilateral cortical regions (Supplementary Figs. 2 and 4). Original labels on the ‘fsaverage’ template cortical surfaces were resampled for each participant’s brain, and the corresponding locations in the functional images were estimated using FreeSurfer. Additionally, the early visual cortex in our study was defined by merging the primary visual cortex (V1) with V2, V3, and V4, which were grouped as early visual cortex in the original parcellation study (Glasser et al., 2016). The cortical region originally named “posterior cingulate” was split into two parts: the precuneus/parieto-occipital sulcus and posterior cingulate cortex based on their differences in canonical dynamics during navigation (Supplementary Fig. 3). The medial temporal lobe was also split into two parts (Supplementary Fig. 4): the hippocampal formation (including the hippocampus proper, presubiculum, and entorhinal cortex) and the parahippocampal region (including parahippocampal area 1,2,3 (area TH), parahippocampal area TF, and perirhinal cortex). All cortical regions were defined for each hemisphere, resulting in a total of 46 regions (23 x 2 hemispheres).

### 2.6 Quantification of spatial memory performance

We assessed participants’ spatial memory performance by calculating the proportion of correct responses to memory questions across all trials. A failure to respond was considered incorrect. Although overall performance was better for the path (mean = 0.719, SE = 0.02) than the place question (mean = 0.655, SE = 0.021) (t(75) = 3.16, p = 0.002), there was a high correlation between the two types of questions (Pearson correlation r = 0.517, p < 0.001). Based on that, we defined an individual’s overall spatial memory performance as the proportion of correct answers across all trials. In one episode, the red arrow indicating the starting position of the navigation was incorrectly marked. Participants’ responses to the question of this particular episode were excluded from the calculation of their spatial memory performance, but their brain activities during the encoding (i.e., watching the navigation episode) were used for the brain data analyses.

### 2.7 Estimation of average activation during navigation

Average activation level (percent signal) was calculated by subtracting the average activity during the two baseline periods (red lines in Fig. 1B) from the navigation phase (except the first 4 frames, blue line in Fig. 1B). The baseline period was set to the last 2 frames (4 seconds) of the two fixation phases. The first few frames were not included to avoid activity related to the prior task, considering the hemodynamic delay. The average activation was calculated for each navigation episode (24) x brain region (46) × participant (76).

### 2.8 Estimation of individual-to-canonical similarity (ICS)

**Fig. 2.**
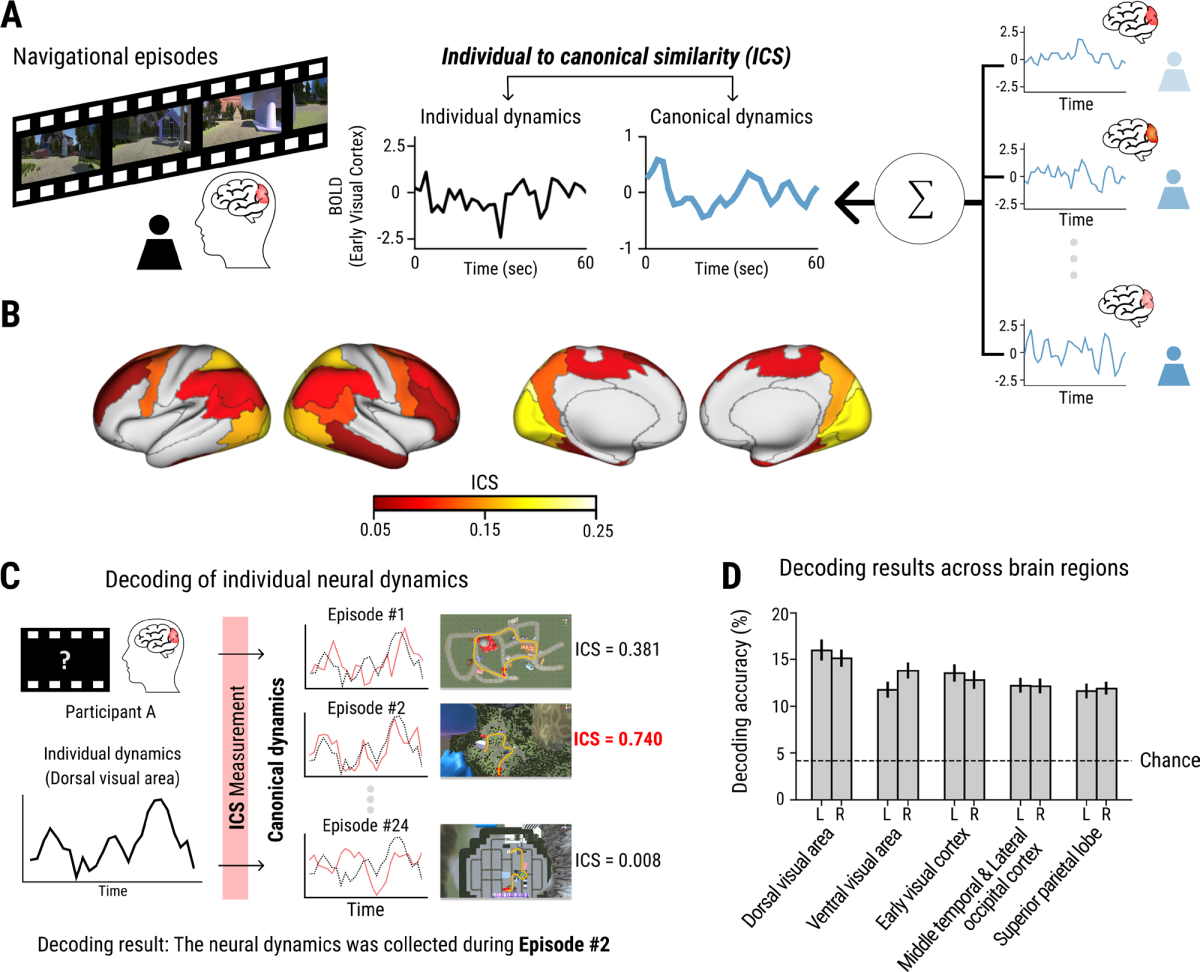
Canonical BOLD dynamics during spatial navigation. (A) Calculation of individual-to-canonical similarity (ICS, see Section 2 for details). (B) ICS was averaged across the 24 navigational episodes and across participants across the whole brain. Only regions with a mean ICS over 0.05 are shown in color. (C) A schematic diagram for decoding navigation episodes from the neural dynamics. ICS of a specific brain region was measured between individual dynamics and canonical dynamics across all episodes, and the experienced episode was predicted as the one with the highest ICS. (D) Average decoding accuracy of the top 10 brain regions. More results are listed in Table 1.

BOLD signals were z-scored across time (for each run; 6 episodes) to avoid the canonical dynamics being disproportionately influenced by individuals with higher variability in their BOLD time series. Then, the ICS was measured by calculating a Pearson correlation between one participant’s dynamics of a region during a specific navigation episode and the average dynamics of that region across the other participants during the same navigation episode (Fig. 2A). This leave-one-subject-out approach was similar to the ones used in previous studies (Nastase et al., 2019; Sheng et al., 2023). The resulting Pearson correlation coefficients were then Fisher Z-transformed, yielding ICS values for each combination of navigation episode (24) x brain region (46) × participant (76).

### 2.9 Estimation of intersubject functional connectivity with the hippocampal formation

Intersubject functional connectivity (FC) with the hippocampal formation was calculated using a Pearson correlation between canonical dynamics of the cortical region Y (averaged across all participants excluding the individual, using the leave-one-subject-out approach) and individual dynamics of the hippocampal formation while watching the same navigation video. The Pearson correlation values were then Fisher Z-transformed. Connectivity was calculated for each navigation episode (24) x cortical region (44) × participant (76). When computing the relationship between connectivity and performance, we used the average connectivity across the 24 navigation episodes.

### 2.10 Decoding navigational episodes from individual neural dynamics

Individual neural dynamics of a specific navigational episode were compared to the canonical dynamics of every episode (total 24) by calculating the Pearson correlation, for each brain region. For this specific analysis, the canonical dynamics were calculated by averaging the time series from every participant except the tested individual (i.e., leave-one-subject-out approach). Among a total of 24 episodes, if one with the highest similarity was identical to the actual navigational episode, the decoding was regarded as successful (or correct) while other cases were regarded as not successful (or incorrect), and thus the chance level was 1/24 (0.041). This procedure was repeated in a leave-one-subject-out fashion for every trial. Consequently, we obtained the decoding results for each participant (76) × trial (24) × brain region (46). The statistical significance of the decoding accuracy was measured by comparing it with the chance level.

### 2.11 Contribution of turn and landmark evoked activities on canonical dynamics

Landmark-viewing periods were characterized using eye-tracking data from a separate group of participants (*n* = 42), who underwent the exact same paradigm but outside the MR scanner. Gaze activities were monitored by an eye tracker (Tobii Pro Fusion) at 250 Hz sampling rate and were resampled to a 10 Hz resolution. The status of landmark viewing at each moment was marked if the participant ratio of gazes on landmarks was above the threshold, which was the top 20% value of the ratio during the 1-minute passive navigation period (see Supplementary Fig. 11). Therefore, 20% of time points in trials with local landmarks were defined as landmark viewing. Turning (during navigation) was measured by the absolute angular velocity of the virtual navigator.

$$Y_{k}\;=\;\beta_{1}X_{1}\;+\;\beta_{2}X_{2}\;+\;i\;+\;e$$ (1)

**Fig. 3.**
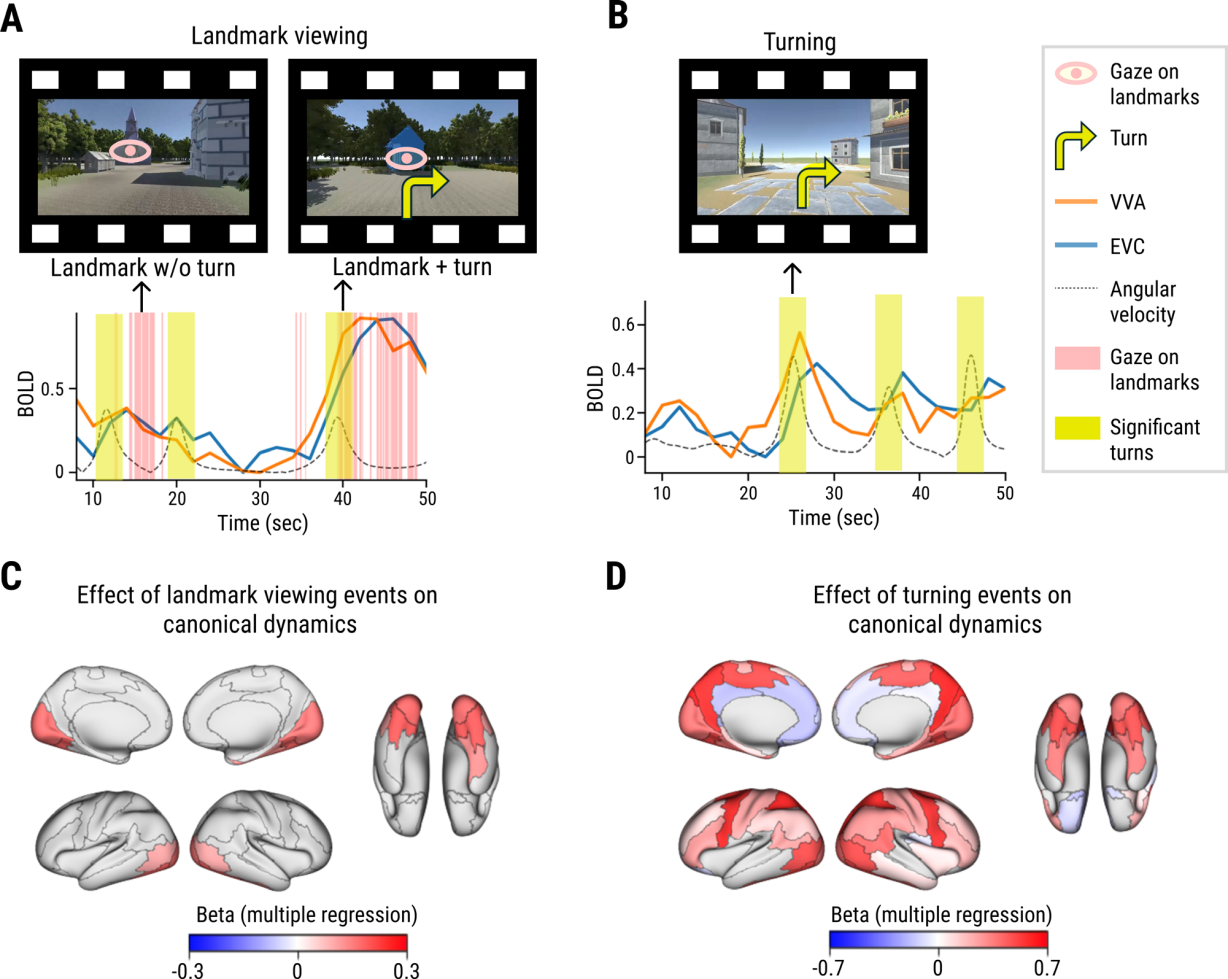
Effects of landmark viewing and turning evoked activities on canonical dynamics. (A) Orange and blue lines indicate canonical dynamics during landmark viewing in the ventral visual area (VVA) and early visual cortex (EVC), respectively. The dotted black line indicates angular velocity of the passive navigation. The yellow shaded areas marks significant turns, and pink vertical lines mark time points when most participants fixated on landmarks. Examples of a landmark viewing event with or without simultaneous turning is indicated by the black arrows, and corresponding movie scenes are shown above. Pink eye icons and yellow turn arrows in sample scenes denote landmark viewing and turning, respectively (for illustration purpose; not present in stimuli). The landmark viewing events were defined by the periods in which participants gazed at local landmarks (See Section 2 for details). (B) An example of a turning event in no landmark condition. (C, D) Coefficients (i.e., beta) for landmark viewing (C) or angular velocity (D) from a multiple regression model describing canonical dynamics of each brain region. Only significant regions (FDR adjusted p < 0.05) are visualized.

The relationship between canonical dynamics and turns or landmark viewing was estimated by a multiple linear regression model (1), in which Y_k_ denotes the canonical dynamics of brain region *k* for all trials, X_1_ denotes the angular velocity (characterizing turns) convolved with the hemodynamic response function, and X_2_ denotes the status of landmark viewing convolved with the hemodynamic response function. In the regression model, *i* and *e* represent the intercept and the error term, respectively. For each passive navigation episode, 26 frames were used (the first 4 frames were excluded, as in the ICS analysis). All variables were standardized by z-scoring before regression. Coefficients β_1_ and β_2_ represent the contributions of turning and landmarks on canonical dynamics, which were visualized on the brain in Figure 3C and 3D.

### 2.12 Effect of age on ICS and intersubject FC

Previous research reported that the alignment of BOLD dynamics to a canonical form, such as individual-to-canonical similarity (ICS), is reduced by head motion. This factor is particularly important to consider when evaluating the influence of age on ICS, as a significant positive correlation was observed between age and participants’ average head motion during the experiment (r = 0.493, p < 0.001). In this context, age-related reductions in ICS may not solely reflect changes in neural dynamics but could also be influenced by increased noise in the BOLD signal due to greater head motion among older participants.

To address this, we estimated the influence of age on ICS using a multiple regression model that included both age and motion as predictors. For standardized effect estimation, ICS, age, and motion values were z-scored across participants:

ICS = a×Age + b×Motion + c + e (2)

Here, *a* is the coefficient for age, *b* is the coefficient for motion, *c* is the intercept, and *e* is the error term. This regression model can be extended into a mediation analysis framework, facilitating further investigation of ICS differences between age groups (e.g., by converting age into a categorical variable), while accounting for motion effects. The variance inflation factor (VIF) for age was 1.32, indicating low multicollinearity and falling well within the acceptable range (VIF < 5). For intersubject FC, a similar analysis was conducted as with ICS.

### 2.13 Mediation analysis

$$Y\;=\;cX\;+\;d_{1}Z\;+\;i_{1}\;+\;e_{1}$$ (3)

$$M\;=\;aX\;+\;d_{2}Z\;+\;i_{2}\;+\;e_{2}$$ (4)

$$Y\;=\;c'X\;+\;bM\;+\;d_{3}Z\;+\;i_{3}\;+\;e_{3}$$ (5)

A mediation analysis (Eq. 3, 4, and 5) was performed to find the neural correlates of age-related spatial memory decline: Y (dependent variable) denotes memory performance, X (independent variable) denotes age, and M (mediator) denotes each region’s ICS or intersubject FC to the hippocampal formation. The total effect without mediation was measured by *c*. The coefficients *a* and *b* represent the effect of each mediation path, and the remaining direct effect was measured by *c*'. Similar to the previous analyses, we considered an individual’s head motion as a confounding variable that needed to be controlled. Confounding variable Z in the regression model represents an individual’s head motion and coefficient *d* represents the contribution of confounding variable to dependent variables, while *i* and *e* represent the intercept and residual of the model. X, Y, M, and Z were standardized (z-scored) before the regression. Before testing the significance of the indirect (mediation) effect, we confirmed that there was a significant total effect (*c* = -0.697, SE = 0.096, p < 0.001) in the first regression model, indicating the spatial memory decline in aging. We also found neural activities (i.e., brain-wide ICS or intersubject FC with the hippocampal formation) that decreased with aging (*a* < 0 in the second model with p < 0.05) in some brain regions and considered these regions as a candidate mediating the age-related spatial memory decline (Fig. 6 and Supplementary Table 1). Finally, we tested whether there was a significant negative indirect (mediation) effect (product of coefficients = *a* * *b*) for each brain region’s ICS or intersubject FC using the Sobel z-test (Sobel, 1982) along with the remaining direct effect between age and performance by *c*’.

### 2.14 Statistical tests

A permutation test was used to determine whether the mean ICS of each cortical region was significantly different from zero (Fig. 2, Table 1). We calculated the correlation between the canonical dynamics of each map and the individual dynamics of a randomly selected map (i.e., permutation) to generate a null distribution. The permutation process was repeated 10,000 times, and the statistical significance of the mean ICS was determined by comparing its value with the null distribution. Similarly, an across-episode permutation test was used to validate the performance of the decoding of a navigational episode based on the canonical dynamics (Fig. 2 and Table 1). We permuted a label of the canonical dynamics and applied the same decoding procedures to generate a null distribution of decoding accuracy in each brain region. The statistical significance of the decoding performance was measured by comparing the actual accuracy with the null distribution.

**Table 1.** Individual-to-canonical similarity (ICS) and decoding accuracy of navigational episodes for each brain region.

		ICS			Decoding performance (%)		
Region	Hemisphere	Mean	SE	p-value	Mean	SE	p-value
Dorsal visual area	(L)	**0.218**	**0.011**	<0.0001*	**16.009**	**1.126**	<0.0001*
	(R)	**0.208**	**0.011**	<0.0001*	**15.132**	**0.923**	<0.0001*
Ventral visual area	(L)	**0.164**	**0.009**	<0.0001*	**11.787**	**0.865**	<0.0001*
	(R)	**0.192**	**0.009**	<0.0001*	**13.816**	**0.847**	<0.0001*
Early visual cortex	(L)	**0.186**	**0.009**	<0.0001*	**13.542**	**0.931**	<0.0001*
	(R)	**0.181**	**0.010**	<0.0001*	**12.829**	**0.977**	<0.0001*
Superior parietal lobe	(L)	**0.169**	**0.008**	<0.0001*	**11.623**	**0.773**	<0.0001*
	(R)	**0.173**	**0.009**	<0.0001*	**11.897**	**0.701**	<0.0001*
Middle temporal area/lateral occipital cortex	(L)	**0.159**	**0.007**	<0.0001*	**12.226**	**0.811**	<0.0001*
	(R)	**0.162**	**0.009**	<0.0001*	**12.171**	**0.805**	<0.0001*
Precuneus/parieto-occipital sulcus	(L)	**0.121**	**0.009**	<0.0001*	**8.827**	**0.842**	<0.0001*
	(R)	**0.152**	**0.009**	<0.0001*	**11.129**	**0.741**	<0.0001*
Premotor cortex	(L)	**0.126**	**0.009**	<0.0001*	**8.333**	**0.644**	<0.0001*
	(R)	**0.122**	**0.010**	<0.0001*	**10.143**	**0.840**	<0.0001*
Temporo-parieto-occipital junction	(L)	**0.084**	**0.009**	<0.0001*	**6.086**	**0.499**	0.0002*
	(R)	**0.113**	**0.009**	<0.0001*	**8.224**	**0.635**	<0.0001*
Inferior parietal lobe	(L)	**0.078**	**0.007**	<0.0001*	**6.579**	**0.561**	<0.0001*
	(R)	**0.091**	**0.008**	<0.0001*	**7.127**	**0.643**	<0.0001*
Dorsolateral prefrontal	(L)	**0.051**	**0.007**	<0.0001*	**5.318**	**0.518**	0.0113*
	(R)	**0.078**	**0.010**	<0.0001*	**5.702**	**0.569**	0.0011*
Paracentral lobule/midcingulate cortex	(L)	**0.073**	**0.007**	<0.0001*	**6.853**	**0.633**	<0.0001*
	(R)	**0.072**	**0.008**	<0.0001*	**7.237**	**0.607**	<0.0001*
Parahippocampal region	(L)	**0.056**	**0.006**	<0.0001*	**7.018**	**0.636**	<0.0001*
	(R)	**0.071**	**0.007**	<0.0001*	**6.524**	**0.561**	<0.0001*
Inferior frontal cortex	(L)	**0.047**	**0.006**	<0.0001*	4.770	0.450	0.1009
	(R)	**0.059**	**0.007**	<0.0001*	**5.976**	**0.539**	0.0012*
Lateral temporal lobe	(L)	**0.033**	**0.006**	<0.0001*	**6.360**	**0.637**	<0.0001*
	(R)	**0.051**	**0.006**	<0.0001*	**5.482**	**0.571**	0.0041*
Posterior opercular cortex	(L)	**0.048**	**0.006**	<0.0001*	4.660	0.559	0.1695
	(R)	**0.049**	**0.006**	<0.0001*	**5.373**	**0.584**	0.0096*
Posterior cingulate cortex	(L)	**0.045**	**0.006**	<0.0001*	**5.976**	**0.498**	0.0002*
	(R)	**0.030**	**0.008**	0.0084*	**4.934**	**0.524**	**0.0453**
Anterior cingulate cortex/medial prefrontal cortex	(L)	**0.035**	**0.007**	<0.0001*	**5.099**	**0.524**	0.0321*
	(R)	**0.045**	**0.008**	<0.0001*	**4.989**	**0.532**	**0.0484**
Auditory association area	(L)	**0.042**	**0.008**	0.0001*	**5.702**	**0.574**	0.001*
	(R)	**0.034**	**0.007**	<0.0001*	**5.263**	**0.516**	0.0116*
Somatosensory/motor cortex	(L)	**0.041**	**0.007**	<0.0001*	**5.099**	**0.475**	0.0266*
	(R)	**0.036**	**0.007**	0.0002*	**5.428**	**0.575**	0.0027*
Early auditory cortex	(L)	**0.029**	**0.007**	0.0014*	4.386	0.458	0.3038
	(R)	**0.040**	**0.006**	<0.0001*	4.660	0.585	0.1495
Insular/frontal opercular cortex	(L)	**0.036**	**0.006**	0.0001*	4.057	0.502	0.5828
	(R)	**0.038**	**0.008**	0.0006*	**6.250**	**0.534**	0.0002*
Orbitofrontal cortex/frontopolar cortex	(L)	**0.032**	**0.006**	<0.0001*	**5.757**	**0.531**	0.0009*
	(R)	**0.021**	**0.006**	0.0004*	4.825	0.491	0.0832
Hippocampal formation	(L)	**0.019**	**0.006**	0.0011*	4.879	0.567	0.0513
	(R)	**0.021**	**0.005**	0.0004*	**5.647**	**0.547**	0.0021*

p-values are uncorrected for each permutation test (see Section 2 for details). The regions are in descending order of ICS. Statistically significant results are presented in bold font (p < 0.05), and asterisks denote significance following multiple comparisons correction (FDR adjusted p < 0.05).

The significance of the contributions of landmark and turn events (Fig. 3, Table 2) was determined by testing whether the coefficients of the multiple linear regression model (Eq. 1) were significantly different from zero using a two-tailed test. The coefficients and p-values were obtained using the ordinary least-squares method (Statsmodels package in Python). The same method was used to determine the significance of age on ICS and intersubject FC by the regression model (Table 5).

**Table 2.** Coefficients of a multiple linear regression model for the canonical dynamics of each region with the time series of landmark viewing and angular velocity, along with their standard error (SE).

		Landmark viewing		Turning		
Region	Hemisphere	b (SE)	p-value	b (SE)	p-value	R-squared
Ventral visual area	(L)	**0.197 (0.034)**	<0.0001*	**0.495 (0.034)**	<0.0001*	0.3204
	(R)	**0.193 (0.034)**	<0.0001*	**0.478 (0.034)**	<0.0001*	0.3005
Early visual cortex	(L)	**0.179 (0.037)**	<0.0001*	**0.350 (0.037)**	<0.0001*	0.1785
	(R)	**0.170 (0.037)**	<0.0001*	**0.375 (0.037)**	<0.0001*	0.1930
Parahippocampal region	(L)	**0.088 (0.037)**	**0.0197**	**0.372 (0.037)**	<0.0001*	0.1581
	(R)	**0.132 (0.037)**	0.0004*	**0.357 (0.037)**	<0.0001*	0.1630
Middle temporal area/lateral occipital cortex	(L)	**0.107 (0.035)**	0.0026*	**0.467 (0.035)**	<0.0001*	0.2481
	(R)	**0.107 (0.035)**	0.0026*	**0.472 (0.035)**	<0.0001*	0.2530
Temporo-parieto-occipital junction	(L)	0.006 (0.039)	0.8726	**0.259 (0.039)**	<0.0001*	0.0676
	(R)	0.065 (0.037)	0.0799	**0.400 (0.037)**	<0.0001*	0.1741
Dorsal visual area	(L)	0.050 (0.032)	0.1145	**0.613 (0.032)**	<0.0001*	0.3902
	(R)	0.055 (0.032)	0.0859	**0.605 (0.032)**	<0.0001*	0.3817
Hippocampal formation	(L)	0.007 (0.041)	0.8539	**0.128 (0.041)**	0.0017*	0.0168
	(R)	0.051 (0.040)	0.2001	**0.241 (0.040)**	<0.0001*	0.0654
Somatosensory/motor cortex	(L)	-0.005 (0.040)	0.8905	**0.253 (0.040)**	<0.0001*	0.0637
	(R)	0.048 (0.039)	0.2279	**0.243 (0.039)**	<0.0001*	0.0656
Posterior cingulate cortex	(L)	0.005 (0.040)	0.8905	**-0.214 (0.040)**	<0.0001*	0.0454
	(R)	0.037 (0.041)	0.3573	**-0.098 (0.041)**	0.0166*	0.0096
Inferior frontal cortex	(L)	0.016 (0.039)	0.6915	**0.267 (0.039)**	<0.0001*	0.0731
	(R)	0.033 (0.040)	0.4092	**0.204 (0.040)**	<0.0001*	0.0450
Paracentral lobule/midcingulate cortex	(L)	-0.008 (0.035)	0.8206	**0.507 (0.035)**	<0.0001*	0.2552
	(R)	0.029 (0.036)	0.4299	**0.459 (0.036)**	<0.0001*	0.2163
Precuneus/parieto-occipital sulcus	(L)	0.022 (0.034)	0.5159	**0.565 (0.034)**	<0.0001*	0.3239
	(R)	0.024 (0.032)	0.4607	**0.602 (0.032)**	<0.0001*	0.3678
Inferior parietal lobe	(L)	0.024 (0.040)	0.5554	**0.152 (0.040)**	0.0002*	0.0250
	(R)	0.012 (0.039)	0.7684	**0.284 (0.039)**	<0.0001*	0.0822
Orbitofrontal cortex/frontopolar cortex	(L)	0.024 (0.041)	0.5463	**-0.123 (0.041)**	0.0024*	0.0147
Dorsolateral prefrontal	(L)	0.014 (0.041)	0.7377	**0.111 (0.041)**	0.0066*	0.0130
	(R)	0.018 (0.040)	0.6554	**0.158 (0.040)**	0.0001*	0.0265
Posterior opercular cortex	(R)	0.008 (0.041)	0.8379	**-0.085 (0.041)**	0.0376*	0.0070
Insular/frontal opercular cortex	(R)	0.004 (0.041)	0.9207	**0.102 (0.041)**	0.0119*	0.0107
Anterior cingulate cortex/medial prefrontal cortex	(L)	-0.056 (0.040)	0.1626	**-0.187 (0.040)**	<0.0001*	0.0419
	(R)	0.007 (0.041)	0.8632	**-0.126 (0.041)**	0.0020*	0.0155
Premotor cortex	(L)	-0.034 (0.033)	0.3073	**0.601 (0.033)**	<0.0001*	0.3545
	(R)	0.006 (0.034)	0.8610	**0.566 (0.034)**	<0.0001*	0.3218
Superior parietal lobe	(L)	-0.015 (0.032)	0.6367	**0.617 (0.032)**	<0.0001*	0.3774
	(R)	-0.016 (0.033)	0.6247	**0.603 (0.033)**	<0.0001*	0.3606

Only cortical regions with significant coefficients (either landmark viewing or turning) are listed in a descending order. p-values are uncorrected, statistically significant results are in bold font, and asterisks indicate significance after multiple comparison correction (FDR adjusted p < 0.05). R-squared values indicate goodness of fit of the multiple linear regression.

**Fig. 4.**
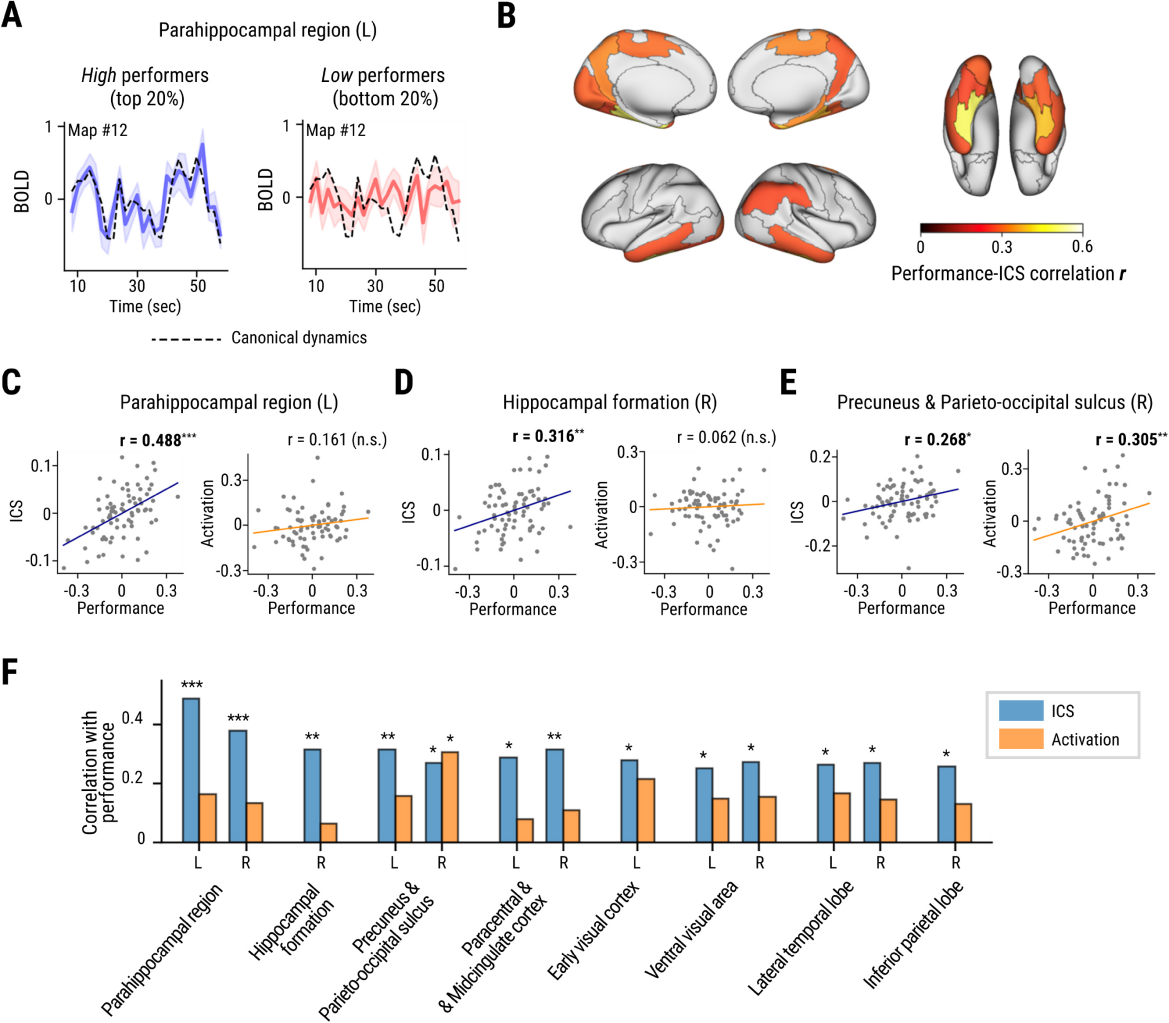
Relationship between individual-to-canonical similarity (ICS) and spatial memory performance. (A) BOLD time series in the left parahippocampal region. The blue line shows average activation in high performers (left, top 20%), and the red line indicates average activation in low performers (right, bottom 20%). Shaded areas indicate standard errors. The black dashed line corresponds to the average of all (N=76) participants (i.e., canonical dynamics). (B) Brain regions in which ICS was correlated with spatial memory performance (p < 0.05). (C–E) Scatter plots for individual spatial memory performance and ICS (left) or activation (right) in the representative regions: the left parahippocampal region, right hippocampal formation, and right precuneus and parieto-occipital sulcus. Individual average head-motion was regressed out for all variables (see Table 3 for other brain regions). Blue and orange lines show the best fit line for each plot. (F) Comparison of average correlation between performance and ICS (blue) and activation level (orange) in brain regions with significant relationships between performance and ICS. *p < 0.05, **p < 0.01, ***p < 0.001.

**Fig. 5.**
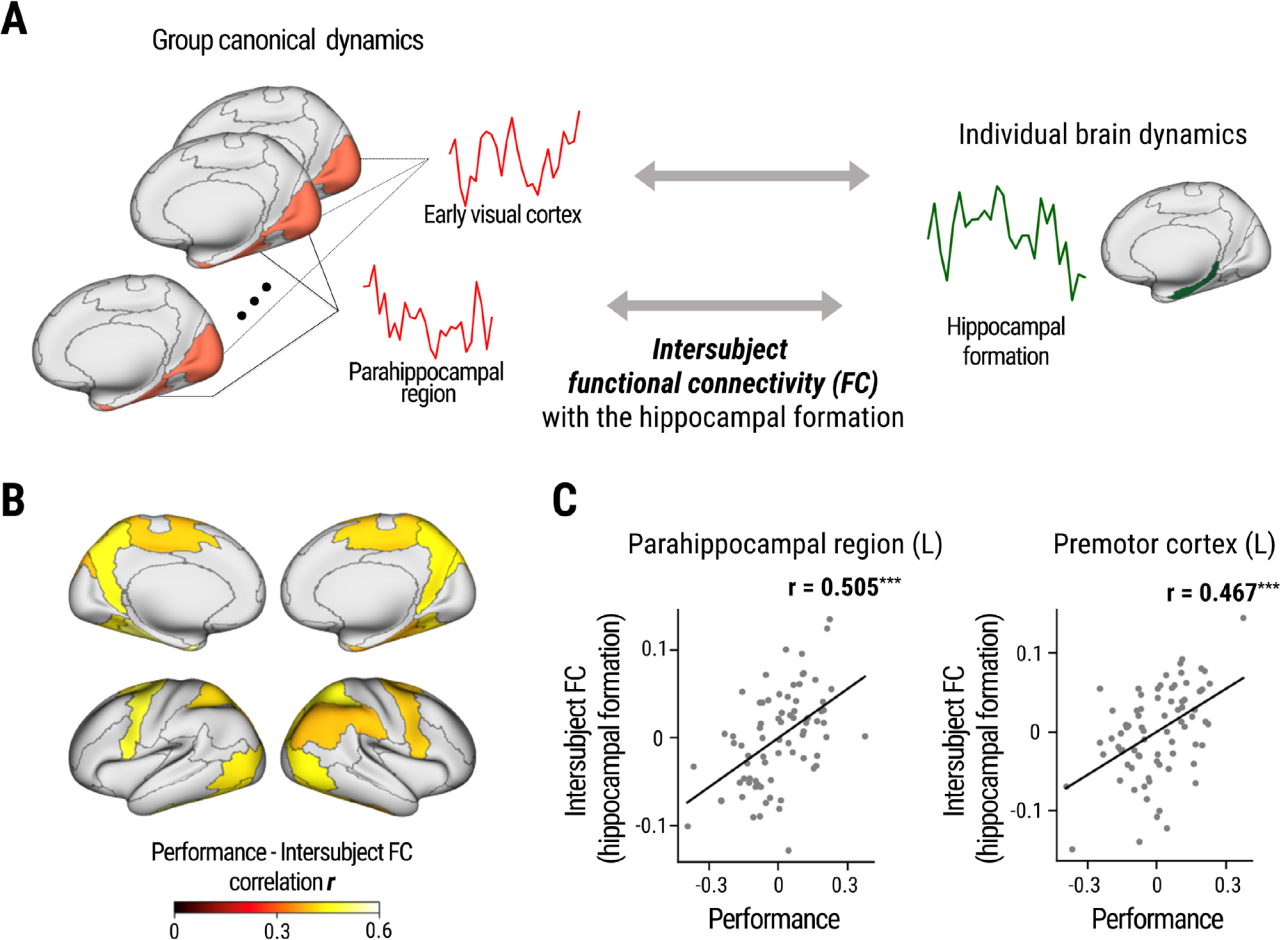
Relationship between intersubject functional connectivity and spatial memory performance. (A) A schematic diagram of intersubject functional connectivity calculated as the Pearson correlation between canonical dynamics of each cortical region and individual hippocampal dynamics. (B) Cortical regions with a higher correlation between intersubject functional connectivity with the hippocampal formation and spatial memory performance are highlighted (p < 0.001). (C) A scatter plot and linear fit lines for individual spatial memory performance and intersubject functional connectivity with the hippocampal formation in two regions after regressing out the effects of motion. ***p < 0.001.

The statistical significance of Pearson correlations was assessed by calculating the p-value for the observed correlation coefficient *r*. Under the null hypothesis of no correlation, *r* follows the probability density function: $f\left(r\right)=\frac{{\left(1-r^{2}\right)}^{\frac{n-4}{2}}}{B\left(\frac{1}{2}\;,\;\;\;\frac{n-2}{2}\right)}$, where *B* denotes the beta function and *n* is the sample size. The p-value was computed using this distribution in a two-tailed test to account for deviations in either direction from the null hypothesis. In the analyses of individual differences (Figs. 4 and 5; Tables 3 and 4) (*n* = 76), the averaged head motion of each individual was regressed out before calculating the Pearson correlation. An individual’s head motion was defined by average framewise displacement (Power et al., 2012).

**Table 3.** Correlations between spatial memory performance and ICS or average activation across brain regions in which ICS or activation showed a positive relationship with performance (p < 0.05).

Correlation with performance		ICS		Average activation	
Region	Hemisphere	Correlation r	p-value	Correlation r	p-value
Parahippocampal region	(L)	**0.488**	7.80 x 10^-6*^	**0.161**	**0.164**
	(R)	**0.377**	0.001*	**0.132**	**0.254**
Hippocampal formation	(R)	**0.316**	**0.005**	**0.062**	**0.595**
Precuneus/parieto-occipital sulcus	(L)	**0.315**	**0.006**	**0.157**	**0.176**
	(R)	**0.268**	**0.020**	**0.305**	**0.007**
Paracentral lobule/mid-cingulate cortex	(L)	**0.288**	**0.012**	**0.079**	**0.496**
	(R)	**0.314**	**0.006**	**0.108**	**0.354**
Early visual cortex	(L)	**0.278**	**0.015**	**0.214**	**0.063**
Ventral visual area	(L)	**0.251**	**0.029**	**0.148**	**0.203**
	(R)	**0.272**	**0.017**	**0.154**	**0.185**
Lateral temporal lobe	(L)	**0.264**	**0.021**	**0.167**	**0.150**
	(R)	**0.269**	**0.019**	**0.146**	**0.209**
Inferior parietal lobe	(R)	**0.256**	**0.026**	**0.131**	**0.261**
Superior parietal lobe	(L)	**0.176**	**0.128**	**0.295**	**0.01**
	(R)	**0.212**	**0.066**	**0.332**	0.003*
Dorsal visual area	(L)	**0.108**	**0.355**	**0.238**	**0.038**
	(R)	**0.072**	**0.535**	**0.242**	**0.035**

Regions are listed in order of correlation between performance and ICS. Significant relationships (either ICS or activation) are shown in bold font, and asterisks indicate significance after multiple comparison correction (FDR adjusted p < 0.05).

**Table 4.** Pearson correlations between each cortical region’s intersubject functional connectivity with the hippocampal formation and spatial memory performance.

Correlation with performance		Intersubject functional connectivity with the hippocampus	
Region	Hemi sphere	Correlation r	p-value
Parahippocampal region	(L)	**0.505**	3.28 x 10^-6*^
	(R)	**0.375**	8.59 x 10^-4*^
Premotor cortex	(L)	**0.467**	2.14 x 10^-5*^
	(R)	**0.401**	3.34 x 10^-4*^
Precuneus/parieto-occipital sulcus	(L)	**0.457**	3.28 x 10^-5*^
	(R)	**0.434**	8.80 x 10^-5*^
Superior parietal lobe	(L)	**0.417**	1.82 x 10^-4*^
	(R)	**0.453**	3.93 x 10^-5*^
Middle temporal area/lateral occipital cortex	(L)	**0.429**	1.09 x 10^-4*^
	(R)	**0.441**	6.68 x 10^-5*^
Ventral visual area	(L)	**0.435**	8.69 x 10^-5*^
	(R)	**0.425**	1.29 x 10^-4*^
Dorsal visual area	(L)	**0.380**	7.16 x 10^-4*^
	(R)	**0.430**	1.06 x 10^-4*^
Paracentral lobule/mid-cingulate cortex	(L)	**0.390**	4.96 x 10^-4*^
	(R)	**0.399**	3.54 x 10^-4*^
Inferior parietal lobe	(R)	**0.383**	6.34 x 10^-4*^

Only cortical regions with highly significant correlations (p < 0.001) are listed in descending order. Statistically significant results are indicated by bold font (p < 0.05), and asterisks indicate significance after multiple comparison correction (FDR adjusted p < 0.05). See Supplementary Table 3 for all results.

## 3 Results

### 3.1 Shared canonical brain dynamics across individuals during navigation

The neural activation dynamics of 46 brain regions (see Supplementary Figs. 2-4) were defined by calculating a time series of average BOLD activity during each navigational episode. To identify regions showing highly similar, canonical temporal dynamics across participants, we calculated individual-to-canonical similarity (ICS) scores by correlating each participant’s time series and the canonical dynamics calculated from the other participants (Fig. 2A and Supplementary Fig. 10; leave-one-subject-out approach). This resulted in ICS values in a combination of 46 cortical regions, 24 navigational episodes, and 76 participants.

Most of individual neural dynamics were aligned to the canonical dynamics as represented by high ICS across the whole brain (Permutation test, FDR adjusted p < 0.05, see Table 1), with the highest ICS found in the visual processing areas in the occipital lobe (Fig. 2B). Additionally, the superior parietal lobe, the premotor cortex, and the parahippocampal region showed a relatively higher ICS (Table 1). In contrast to the highly time-locked shared dynamics in the visual processing regions in the posterior and parietal regions of the cortex, the hippocampal formation and anterior frontal regions such as the orbitofrontal and frontopolar cortex were generally less temporally aligned across participants, resulting in their low ICS.

If neural activation dynamics reflects the processing of important spatial information, the representational neural signature of each navigational episode should be distinguishable from the others. We tested whether the canonical neural dynamics from other participants could be used to decode which navigational episode a particular individual’s neural data were associated with (Fig. 2C). ICS across the 24 episodes in each participant was calculated and regarded as correct if an episode with the highest ICS was identical to the actual episode. Most brain regions showed a statistically significant decoding accuracy above chance level (FDR adjusted p < 0.05 in 37 out of 46 regions; Table 1). In general, brain regions with higher ICS showed greater decoding accuracy; for example, the left dorsal visual area showed the highest ICS and decoding accuracy as expected, based on its well-known role in scene and spatial processing (Goodale & Milner, 1992) (Fig. 2C, 2D). These results confirmed the hypothesis that canonical dynamics during navigation in most of the regions are episode-specific.

### 3.2 Landmark viewing and turning events contribute to canonical dynamics of navigational episodes

If canonical dynamics are determined by the spatial information contained in each episode, then the canonical dynamics during navigational episodes may be aligned to the timing of turning and landmark viewing events (Fig. 3). We measured the degree of turning by calculating angular velocity of the virtual navigation and the extent of landmark viewing by the ratio of participants gazing at the local landmarks at specific timepoints (more details in Section 2). Then, we performed a multiple linear regression to predict the canonical dynamics of each region with the time series of landmark viewing and angular velocity convolved with the hemodynamic response function.

First, a positive association between the canonical dynamics and landmark viewing was found in the ventral visual stream (e.g., bilateral ventral visual area, middle temporal area/lateral occipital cortex, and right parahippocampal region) (Fig. 3A, 3C; Table 2). This functional pathway, especially the parahippocampal place area and lateral occipital cortex, is known for processing scenes and landmarks for spatial mapping (R. Epstein et al., 1999; S. Park et al., 2011). Moreover, in these regions, ICS and decoding accuracy were higher for navigational episodes occurring in environments containing landmarks (Supplementary Figs. 5 and 6), which again supports the hypothesis that neural activities evoked by landmarks were commonly shared across participants. For examples, the decoding accuracy was higher in episodes with landmarks in the ventral visual area (two-sample t-test: t(10) = 2.606, p = 0.026), parahippocampal region (t(10) = 2.503, p = 0.031), and middle temporal area/lateral occipital cortex (t(10) = 2.921, p = 0.015) (Supplementary Fig. 6).

Second, the canonical dynamics in 31 regions (out of 46, Table 2) showed a significant positive relationship with angular velocity (FDR adjusted p < 0.05), time-locked to the turning events (Fig. 3B, 3D). The superior parietal lobe, dorsal visual area, precuneus/parieto-occipital sulcus, and premotor cortex—regions involved in spatial processing (Jerde & Curtis, 2013), attention, and attentional shifts (Corbetta & Shulman, 2002; de Haan et al., 2008)—showed a higher association with the turning events. In contrast, the posterior cingulate cortex, anterior cingulate cortex/medial prefrontal cortex, and right posterior opercular cortex showed a negative association with angular velocity (Fig. 3D), which indicated that turning events induced deactivation in these areas. Given that these regions are a part of the default mode network (e.g., medial prefrontal cortex and posterior cingulate cortex), their suppression may be associated with active task engagement (Fox et al., 2005; Raichle et al., 2001).

In sum, landmark viewing and turn events contributed to the activation dynamics across a broad range of cortical regions (see Supplementary Figs. 5, 6, and 7 for more details). In particular, in the ventral regions of the brain, both landmarks and turns influenced the canonical dynamics, and neither landmark viewing nor turning alone can fully account for these dynamics (Fig. 3A, 3B).

### 3.3 Similarity to canonical dynamics predicts individual spatial memory performance

Given the individual variability in ICS, we tested whether the degree to which the neural dynamics of individuals were well-aligned with the canonical dynamics was correlated with spatial memory accuracy (Fig. 4A). We tested the correlation between ICS of each participant averaged across 24 navigational episodes and their average performance in the spatial memory task in each brain region (Fig. 4B), while regressing out head motion to rule out its potential contribution to the ICS-performance correlation (see Section 2 for details).

The strongest positive relationship between spatial memory and ICS was found in the bilateral parahippocampal region (Fig. 4C) (FDR adjusted p < 0.05). Other regions showing a positive relationship (p < 0.05) were the right hippocampal formation (Fig. 4D), medial and ventral parts of the temporal lobe, and the medial part of the parietal lobe (except the posterior cingulate cortex) (Fig. 4F and Table 3). The association of the MTL regions with performance was indicative of their role in processing the dynamic changes in spatial information during navigation. Interestingly, despite their high ICS overall, the canonical dynamics of brain regions related to low-level visual processing and attention, such as the dorsal visual area (Mishkin et al., 1983) and the superior parietal lobe (Corbetta et al., 1995), were not related to individual memory performance.

Contrary to the neural dynamics, only a few regions showed a positive relationship between average activation and performance (Fig. 4F and Table 3): the bilateral superior parietal lobe, bilateral dorsal visual area, and right precuneus/parieto-occipital sulcus (Fig. 4E). Interestingly, although the hippocampal formation and parahippocampal region showed significant correlations between their canonical dynamics and spatial memory performance, they did not show such correlations in their average activation level. These results indicate heterogeneous roles across brain regions in spatial navigation, with some regions (e.g., precuneus) showing their engagement in continuous spatial updating based on visual stimuli (Wolbers et al., 2008), and other regions (e.g., the visual stream, hippocampus etc.) dynamically time-locked to spatial processes such as turning and landmark viewing.

### 3.4 Correspondence between individual hippocampal dynamics and cortical canonical dynamics predicts memory performance

One possible mechanism underlying individual variability in the hippocampal dynamics may involve the transfer of information from perceptual regions to the hippocampal formation. We tested this hypothesis by calculating the functional connectivity between individual hippocampal dynamics and the canonical dynamics in the other cortical regions (Fig. 5A). This intersubject FC (Simony et al., 2016) indicated how well the visual scene and dynamic spatial information in the cortical canonical dynamics were reflected in the hippocampus for navigational memory encoding.

We found that intersubject FC between the bilateral hippocampal formation and broader cortical regions, including the precuneus and the left parahippocampal region, was strongly correlated with spatial memory performance (Fig. 5B, 5C; Table 4). We also found that hippocampal ICS was correlated with intersubject FC with these regions (Supplementary Fig. 8 and Supplementary Table 3) verifying that the hippocampal activity is dynamically coupled to the cortical visual stream. Additionally, the connectivity with the left premotor cortex and superior parietal cortex also showed a significant correlation with spatial memory performance (Fig. 5B, 5C; Table 4).

When we conducted a similar analysis using other regions of the brain as the seed region, most intersubject FC pairs showed positive correlations, indicating that spatial memory performance improves as the similarity increases between a specific region’s individual (participant-level) dynamics and the canonical dynamics of other regions. These results support the idea of a general effect of neural synchronization on memory. Notably, the relationship between memory performance and intersubject FC with other cortical regions was strongest when the hippocampal formation was used as the seed region (mean r = 0.296; See Supplementary Fig. 9). This may be due to the navigational nature of the task.

### 3.5 Age-related spatial memory decline is mediated by a decrease in similarity to canonical dynamics

**Fig. 6.**
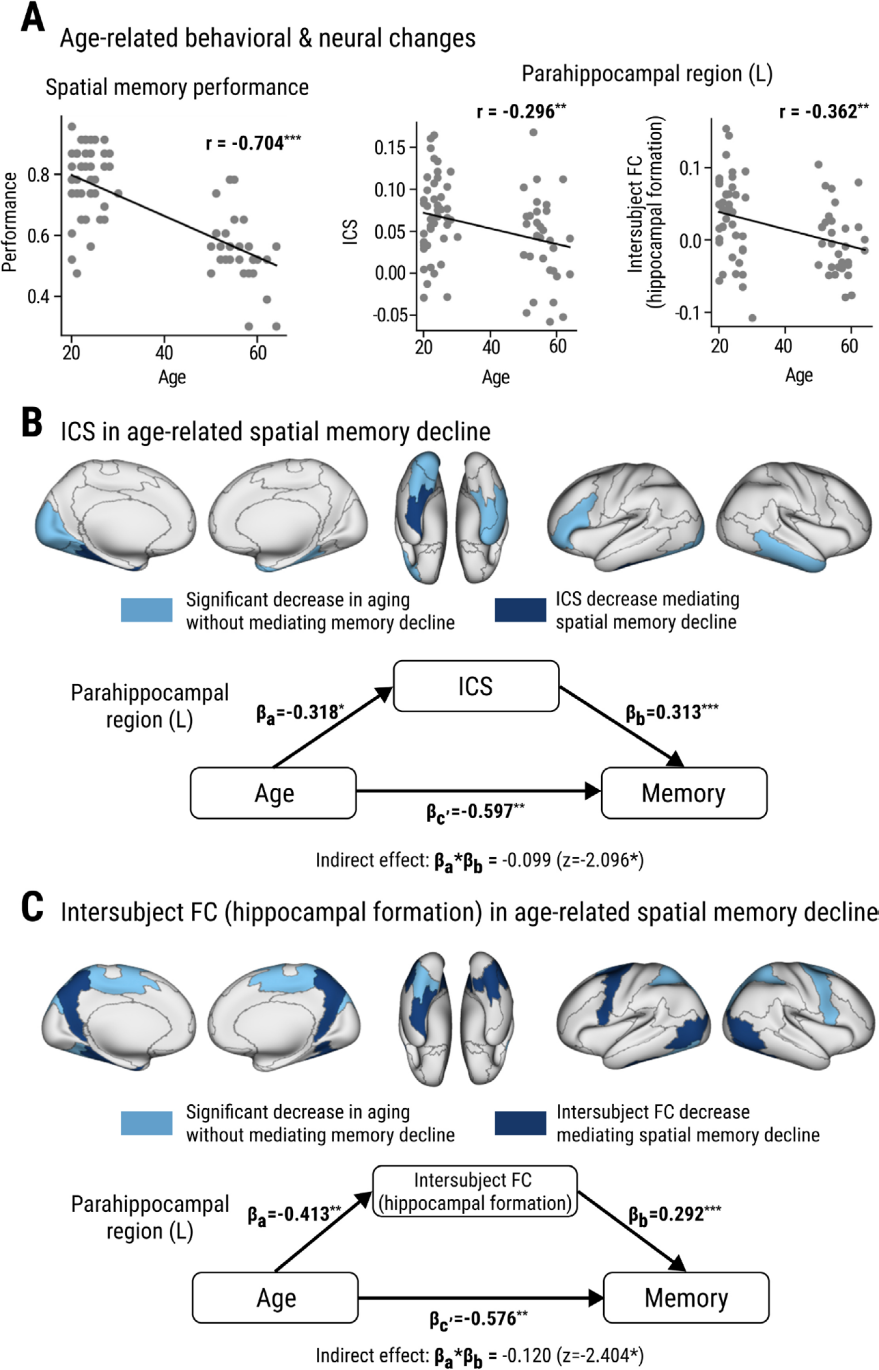
Age-related changes in ICS and intersubject FC. (A) Age-related spatial memory decline (left) and an example of ICS and intersubject FC decline in the left parahippocampal region (see Table 5 for the whole list). (B) *Top*: Shaded brain regions show significant negative correlation between age and ICS (parahippocampal region, ventral visual area, etc.; see Table 5) and dark shaded regions show significant mediation effects (parahippocampal region) of spatial memory decline after individual head motion was regressed out. *Bottom*: The left parahippocampal region shows both mediation of ICS on age-related decline in memory and direct effects of age on memory. (C) *Top*: Shaded brain regions show significant negative correlation between age and the hippocampal intersubject FC (left superior parietal lobe, dorsal visual area, etc.), and dark shaded regions show significant mediation effects (superior parietal lobe, parahippocampal region etc.; see Table 5) of spatial memory decline after individual head motion was regressed out. *Bottom*: A mediation analysis of the left parahippocampal region shows both mediation of hippocampal intersubject FC on age-related decline in memory and direct effects of age on memory. *p < 0.05, **p < 0.01, ***p < 0.001.

Converging upon previous studies reporting spatial memory decline in early aging (Moffat et al., 2001), we found a significant negative correlation between age (range: 20-65) and spatial memory performance (Fig. 6A, Pearson correlation r = -0.704, p < 0.001). Given the significant association between ICS and spatial memory performance in the parahippocampal region (Fig. 4B), we hypothesized that a divergence from the canonical dynamics in the aging brain, especially in this region, may be an indicator of memory decline. To estimate the effects of aging on the ICS of the parahippocampal region of the brain, we conducted a regression analysis while considering the increased head-motion in aging participants (see Section 2.12 for details). An age-related decrease in ICS was found in both left (a = -0.318, p = 0.016) and right (a = -0.275, p = 0.034) parahippocampal regions (Fig. 6A, Table 5). This deviation from the canonical dynamics indicates that individual variability in the dynamic processing of navigational information in the parahippocampal region became more pronounced in aging.

**Table 5.** Regression coefficients with ICS (*top*) and hippocampal intersubject FC (*bottom*) as the dependent variables.

Aging effect on ICS						
		Age regression model		Mediation effects		
Region	Hemisphere	Coefficient	p-value	Indirect effect	Sobel z	p-value
Parahippocampal region	(L)	**-0.3181**	**0.0156**	**-0.0995**	**-2.0959**	**0.0361**
	(R)	**-0.2748**	**0.0344**	-0.0610	-1.6682	0.0953
Ventral visual area	(L)	**-0.2844**	**0.0221**	-0.0243	-0.8639	0.3877
Early visual cortex	(L)	**-0.2709**	**0.0270**	-0.0330	-1.1357	0.2561
Lateral temporal lobe	(R)	**-0.2652**	**0.0431**	-0.0310	-1.1320	0.2576
Inferior frontal cortex	(L)	**-0.2624**	**0.0489**	-0.0158	-0.6615	0.5083
Posterior opercular cortex	(R)	**0.3147**	**0.0175**	0.0337	1.1084	0.2677

Aging effect on intersubject FC with the hippocampal formation						
		Age regression model		Mediation effects		
Region	Hemisphere	Coefficient	p-value	Indirect effect	Sobel z	p-value
Superior parietal lobe	(L)	**-0.4674**	0.0002*	-0.0786	-1.6381	0.1014
	(R)	**-0.4878**	0.0001*	-0.0985	-1.9511	0.0510
Premotor cortex	(L)	**-0.4687**	0.0003*	**-0.1060**	**-2.1304**	**0.0331**
	(R)	**-0.4204**	0.0012*	-0.0740	-1.7246	0.0846
Dorsal visual area	(L)	**-0.3762**	0.0027*	-0.0655	-1.6339	0.1023
	(R)	**-0.4610**	0.0003*	-0.0868	-1.8425	0.0654
Parahippocampal region	(L)	**-0.4125**	0.0015*	**-0.1205**	**-2.4042**	**0.0162**
MT/LOC	(L)	**-0.3760**	0.0030*	**-0.0859**	**-1.9840**	**0.0473**
	(R)	**-0.4053**	0.0016*	**-0.0919**	**-2.0399**	**0.0414**
Ventral visual area	(L)	**-0.2866**	**0.0260**	-0.0791	-1.8758	0.0607
	(R)	**-0.3714**	0.0045*	**-0.0834**	**-1.9658**	**0.0493**
Paracentral/MCC	(L)	**-0.3432**	0.0090*	-0.0691	-1.7778	0.0754
	(R)	**-0.3623**	0.0059*	-0.0731	-1.8212	0.0686
PCun/POS	(L)	**-0.3432**	0.0078*	**-0.0936**	**-2.0942**	**0.0362**
	(R)	**-0.3487**	0.0081*	**-0.0852**	**-1.9978**	**0.0457**

Mediation effects (i.e., a multiplication between two coefficients on the indirect path), their transformation into Sobel z, and the p-values from the Sobel test are listed. Statistically significant results (p < 0.05) are indicated in bold font, and asterisks indicate statistical significance after multiple comparison correction (FDR adjusted p < 0.05).

Next, we conducted a mediation analysis to determine whether spatial memory decline in aging can be, in part, attributed to the age-dependent decrease in ICS in the parahippocampal region. Indeed, the results revealed a significant association between age and performance, mediated by the age-related changes in ICS of the parahippocampal region (left: p = 0.036, right: p = 0.095). We applied this age-related analysis to all other brain regions (Fig. 6B). There was a negative relationship between spatial memory performance and ICS of some brain regions such as the left ventral visual area, right lateral temporal lobe, left inferior frontal cortex, and left early visual cortex (p < 0.05, Table 5). But none of these regions showed a significant mediating effect on age-related performance decline (Table 5; more details in Supplementary Table 1).

Next, we hypothesized that dynamic information transfer between the cortical regions to the hippocampal formation becomes less effective in aging. First, an age-related decline in intersubject FC was found in most of the regions that were associated with spatial memory performance, such as the parahippocampal region (Figs. 5B, 6A, 6C), bilateral superior parietal lobe, and premotor cortex (other regions listed in Table 5). Moreover, the age-related spatial decline was more severe in participants whose hippocampal intersubject FC was lower in these regions (Table 5). This effect was particularly stronger in the left parahippocampal region (Fig. 6C) and the left premotor cortex, although ICS in the premotor cortex was not directly associated with spatial memory decline (Table 5).

Additionally, we found that the regions in which intersubject FC mediated age-related memory decline were visual regions such as the middle temporal lobe/lateral occipital cortex, and dorsal visual area, which were all strongly involved in the turning events (p < 0.05) (Fig. 3D, Table 2). This raises the possibility that aging reduces the communication of turn-evoked neural dynamics of the cortex to the hippocampal memory network. Moreover, previous studies suggested that turns during navigation function as event boundaries (Brunec et al., 2020) and that there is an age-related decline in hippocampal responses at event boundaries (Reagh et al., 2020). Based on those findings, weaker processing of turns as event boundaries may have additionally contributed to lower spatial memory performance in aging individuals.

## 4 Discussion

In this study, we presented a novel method for studying the neural correlates of navigational memory encoding using canonical dynamics that can serve as an alternative to measuring time-averaged activation. We found that shared neural dynamics across participants (i.e., canonical dynamics) aligned to turns and landmark viewing were typically greater in the visual stream and scene-processing regions, reflecting their role in the perception of relevant spatial information. Such dynamic representations were unique to each episode, making it possible to decode the videos from other participants’ neural activity. Furthermore, consistent with the critical role of the MTL in navigation and spatial representation, ICS in the hippocampal and parahippocampal region were strongly associated with spatial memory performance, demonstrating their engagement in spatial mapping. This was confirmed by the strong association between memory performance and dynamic cortico-hippocampal coupling, which declined in aging.

### 4.1 Dynamic and persistent neural activities during spatial navigation and memory encoding

Despite past studies reporting that larger hippocampal activation (and volume) is correlated with better spatial memory (Guderian et al., 2015; Hartley et al., 2003; Wolbers et al., 2007; Xu et al., 2010), it is often the case that the average hippocampal fMRI BOLD signal during navigation does not increase relative to the baseline period (Ekstrom & Isham, 2017; Hartley et al., 2003; Janzen & Jansen, 2010; Rosenbaum et al., 2004). In fact, some studies reported overall activation increases in other brain regions such as the parietal cortex instead (R. A. Epstein et al., 2007; Mitchell et al., 2018). Similar to these past results, we observed an association between average activation and spatial memory performance in the precuneus but not in the hippocampus. Nevertheless, we found that activation dynamics in the hippocampus and its functional connectivity to the cortical canonical dynamics showed a strong association with spatial memory performance.

Dynamic and persistent components of neural activity offered different insights into cognitive processes underlying navigation. Dynamic activation aligned to spatial events such as turning or landmark viewing was observed widely across the brain; the degree to which the MTL followed the canonical cortical dynamics was also critical to spatial memory. The effect of landmarks and turning accords with past studies demonstrating the role of landmarks for building hippocampal cognitive maps (Burgess, 2008; Ekstrom & Ranganath, 2018; Iaria et al., 2007) and the effect of turning during navigation in hippocampal event segmentation (Baldassano et al., 2017; Barnett et al., 2024; Brunec et al., 2020; Reagh et al., 2020). Complementary to dynamic responses, overall activation may signify the continual engagement of certain cognitive processes throughout the entire navigational episode. For instance, the correlation between memory performance and overall activation level of the medial parietal cortex may be attributed to the allocation of spatial attention and the egocentric-to-allocentric translation of spatial information during navigation. Thus, there may be a canonical way in which both dynamic and stable responses are produced by the brain that are important for optimal memory encoding.

Our results align with previous studies showing that dynamic neural responses to complex, naturalistic stimuli are linked to task performance (Chamberlain et al., 2024; Hasson et al., 2008; Jangraw et al., 2023; Sheng et al., 2023). We believe that the current results likely reflect processes that may be more navigation-specific than those typically engaged by general movie-watching. This is because we used first-person perspective in virtual navigation that included naturalistic speed and directional changes as in real-world navigation. Additionally, participants were not merely passively viewing the visual stimuli, and they were actively engaged in a task that involved monitoring and encoding their trajectory and location during navigation. Future studies could explore whether the current effects are, indeed, specific to navigation-related processes or reflect broader mechanisms of dynamic information processing. This could be addressed by comparing conditions such as third-person-view videos (e.g., observing moving agents) or tasks unrelated to navigation (e.g., counting the number of encountered buildings).

### 4.2 Transfer of dynamic information processing between the cortex and the hippocampus underlies memory performance

ICS in the hippocampal formation was lower than many cortical regions of the brain, suggesting that there were greater individual differences in hippocampal dynamics during navigation. According to our findings, the reason for these individual differences lies, in part, in the degree to which the hippocampus reliably entrains with the visuospatial information processes reflected in the cortical dynamics. Indeed, individuals whose hippocampal dynamics were similar to the cortical canonical dynamics, indicated by a higher intersubject FC, performed better in the spatial memory task. Interestingly, our results showed that it is important for the hippocampus to reflect activities not only from the low-level visual processing areas but also the premotor cortex and superior parietal lobe. Both regions showed a higher ICS and tight alignment with turning events, indicating their role in selective attention for processing important information during spatial navigation (Jerde & Curtis, 2013) through their functional coupling to the hippocampus (Kim & Lee, 2023; Simon et al., 2002).

### 4.3 Suboptimal activity dynamics in the MTL mediate age-related spatial memory decline

When the coherence between the hippocampus and cortical dynamics falls apart in aging, it may lead to difficulties in forming detailed memories of a navigational episode. Consistent with existing literature on the pronounced decline in the cortico-hippocampal memory function in the aging brain (Cansino, 2022; Nyberg, 2017; Stark et al., 2021), we found that the parahippocampal region plays an important role in this process. Structurally, the parahippocampal region decreases both in volume (Pantel et al., 2003) and connectivity to the hippocampus (Stark et al., 2021) across aging. Functionally, recent fMRI studies show that aging reduces the parahippocampal region’s sensitivity to spatial contextual information and that this diminished specificity is associated with lower memory performance (Koen et al., 2019, 2020). This alteration in the brain may disturb the incorporation of landmarks and scenes into a detailed spatial map-like representation in memory (Moffat & Resnick, 2002; Sun et al., 2021).

Furthermore, we found that hippocampal connectivity to cortical regions such as paracentral/premotor cortex (i.e., Brodmann area 6), precuneus, and cingulate cortex also mediated the age-related spatial memory decline. These regions have been previously implicated in sequence processing in navigation and episodic memory (Carpenter et al., 2018; Hwang & Lee, 2024; Kim & Lee, 2023) and the particular vulnerability of scene order memory in aging (J. H. Park & Lee, 2021; S. E. Park et al., 2023).

Given the age-related loss in hippocampal synaptic plasticity (Burke & Barnes, 2006; Foster, 1999; Shen et al., 1997), we speculate that an age-related decline in cortico-hippocampal network dynamics reflects a reduction in the ability of the hippocampus to modulate its activity in accord with the relevant input from the cortex. This seems to be the case for both bottom-up and top-down processes, given the importance of its coupling with regions implicated in attention, perception, and sequence processing.

Our approach to investigating age-related changes in dynamic brain activity during memory encoding offers new insights that may be complementary to the results of previous fMRI studies which mostly focused on conventional measures of BOLD activation levels. Because aging affects non-neuronal factors such as vascular changes, which can also reduce the overall level of the BOLD signals (Buckner et al., 2000; Hesselmann et al., 2001; Ross et al., 1997), it poses an additional challenge to interpret age-related changes during complex cognitive tasks as evidence of reduced engagement of different brain regions. In contrast, changes in ICS are independent from the activation level and, therefore, provide a normalized metric for capturing suboptimal neural responses in aging individuals.

### 4.4 Limitations and future studies

In this study, we characterized the canonical neural dynamics across the whole brain while participants were passively navigated around virtual environments. Measuring neural activity in this way was particularly beneficial for this paradigm because the spatial episodes were identical across every participant. However, our findings may not be entirely generalizable to an active navigation paradigm or to real navigation involving vestibular and proprioceptive information. Moreover, virtual environments that are artificially created (e.g., Unity) may be more familiar to some participants (younger participants) than to others (older participants). This means that individual differences in task performance and neural dynamics may reflect not only spatial memory ability but also familiarity with the type of stimuli we used. We expect that using more realistic stimuli and including pre-training sessions could help minimize such effects.

Another limitation is related to the age range of our participants. Despite finding a significant age-dependent decrease in task performance and related neural correlates (ICS and intersubject FC) in the MTL, the older participant group in our study (50-65 years) is relatively young compared to those typically included in cognitive aging research (Nyberg et al., 2012). Also, the absence of participants aged 31 to 49 in our dataset makes it less ideal to treat age as a continuous variable in the main analysis (for results using age as a group variable, see Supplementary Table 2). Therefore, future studies are needed to fill these gaps to determine whether the decreases in performance and neural dynamics observed here can be generalized across the adult lifespan (e.g., from 20 to 90).

Finally, given that the neural dynamics is time-locked to the presentation of significant information within a navigational episode, our approach may not be optimal for memory tasks using static stimuli such as scenes. Unlike extended videos, scenes do not provide salient temporal dynamics and thus may induce individual variances in the exact timing of information processing across the presentation phase, making their canonical neural dynamics difficult to capture. In such cases, average activation levels may be a better measure for measuring the engagement of brain regions across entire periods of memory encoding.

### 4.5 Conclusion

Through the use of a controlled virtual navigation task, we have demonstrated that canonical dynamics represent the brain’s optimal neural processing of information across a temporally extended spatial event. Moreover, we revealed that the translation of visual stimuli to navigational episodic memory is particularly reliant on a cortico-hippocampal network, which declines in aging. The results highlight the importance of dynamic neural analyses in studies of spatial navigation alongside measures of overall brain activation.

## Supplementary Material

Supplementary Material

## Data Availability

Data and code can be downloaded from following location. https://osf.io/fyuj4/?view_only=1ff30e6431d74bbc8678620ac2e0078b
