# Supplementary material for: Canonical cortico-hippocampal dynamics underlie memory of navigational episodes and its early decline in aging
Source: Imaging Neurosci (Camb). 2025 Aug 18;3:IMAG.a.101. doi: 10.1162/IMAG.a.101 (PMC12362303; doi:10.1162/IMAG.a.101)
Supplement: Supplementary Material [file IMAG.a.101_supp.pdf]

Supplementary Material

Supplementary Figure 1. An example of the overhead-view maps in four environments used in this study.

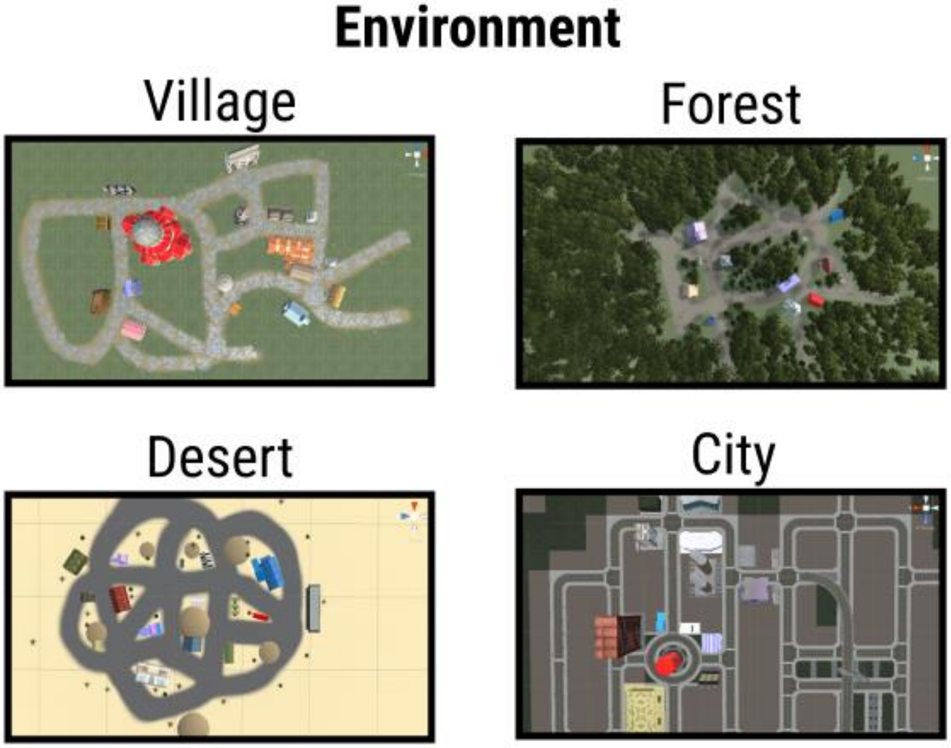

We developed 24 navigational episodes in 4 different environments (village, forest, desert, and city). The forest and city environments included two episodes in “Landmarks” and “Landmarks & Distal Cues” conditions each and one episode in “Distal Cues” and “No Landmarks” conditions each. The village and desert environments included one episode in “Landmarks” and “Landmarks & Distal Cues” conditions each and two episodes in “Distal Cues” and “No Landmarks” conditions each.

Supplementary Figure 2. Brain region grouping based on human connectome project multimodal parcellation

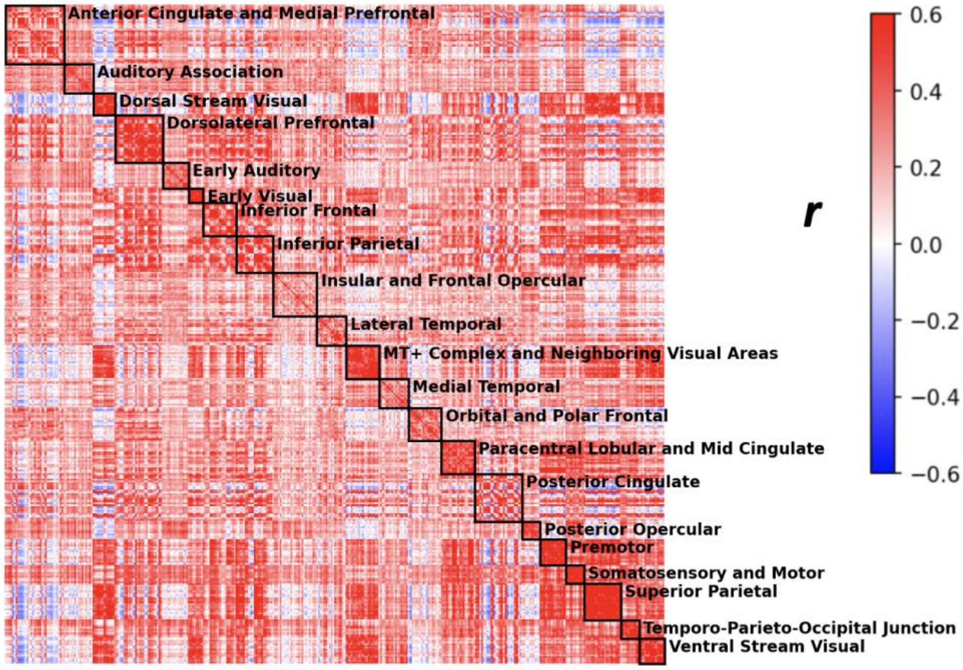

Brain regions were segmented or merged based on their anatomical proximity and functional similarity. First, the Human Connectome Project Multimodal Parcellation (HCP MMP; (Glasser et al., 2016)) provided 360 subregions grouped into 23 larger divisions (Glasser et al., 2016; Huang et al., 2022). Then we produced a correlation matrix that represents the similarity of canonical dynamics (Fig. 2) between subregions. This was done by calculating a Pearson correlation between averaged BOLD dynamics (across participants) of two regions, then averaging the correlations across all navigation episodes. Most subregions within the same division exhibited similar canonical dynamics, as indicated by positive correlation values within the black squares, suggesting that each division can be treated as a single functional "unit" for processing navigation episodes. Therefore, these brain segments on each hemisphere were used for all analyses in this study. We made three changes from the original grouping: First, the primary visual cortex (V1) and early visual cortex (V2, V3, V4) were merged into a single group, referred to as the 'early visual cortex,' based on their similar canonical dynamics and anatomical locations. Two additional changes were in the posterior cingulate cortex and the medial temporal lobe, which are described below.

Supplementary Figure 3. Dividing the posterior cingulate into two parts: the precuneus/parieto-occipital sulcus and the posterior cingulate cortex

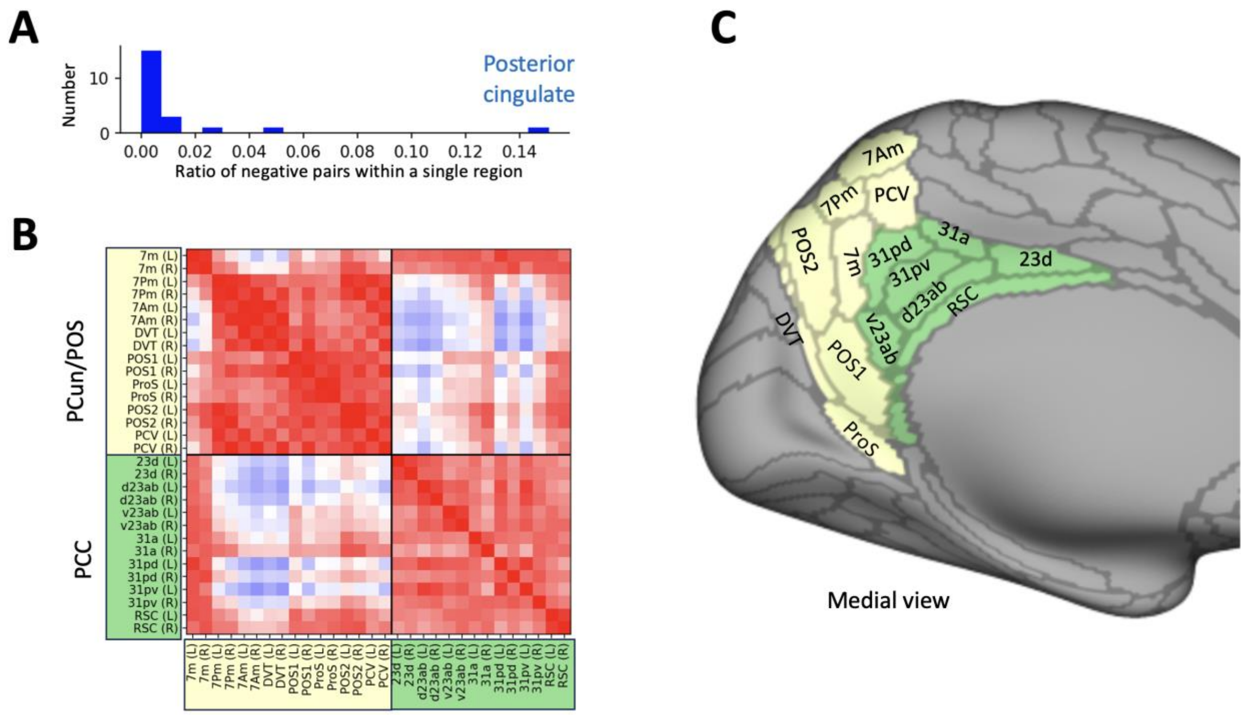

A. Compared to the other divisions, the posterior cingulate showed a high ratio of negative pairwise correlations indicating inhomogeneous canonical dynamics across its subregions, prompting its split into two parts to increase the within-region homogeneity in neural activities.

B and C. The first part included the precuneus (PCun; consisting of medial part of BA 7, PCV; all subregions were based on the HCP MMP) and the parieto-occipital sulcus (POS; consisting of dorsal visual transfer region, prostriate, POS1, and POS2) (Baker et al., 2018; Yamaguchi & Jitsuishi, 2023). Subregions Area 7Pm and 7Am were originally part of the superior parietal division, but we included them in the PCun/POS due to their anatomical proximity with the dorsal part of the precuneus (Yamaguchi & Jitsuishi, 2023). The other part, referred to as the posterior cingulate cortex (PCC) in our study, included retrosplenial complex (RSC), BA 23, and BA 31 (Rolls et al., 2023)

Although the RSC was grouped with the PCC based on anatomical proximity, it also showed a similar neural pattern with the PCun/POS. Similarly, one of subregions of the PCun/POS, the ventral bank of the parieto-occipital sulcus (POS1), was occasionally interpreted

as a part of RSC (Nasr et al., 2011; Silson et al., 2016) based on its significant scene-selective activation (Glasser et al., 2016). However, the results of our study were not significantly changed by a categorization of the RSC as either PCC or PCun/POS.

Supplementary Figure 4a. Split the medial temporal lobe into two parts: the Hippocampal formation and the parahippocampal region

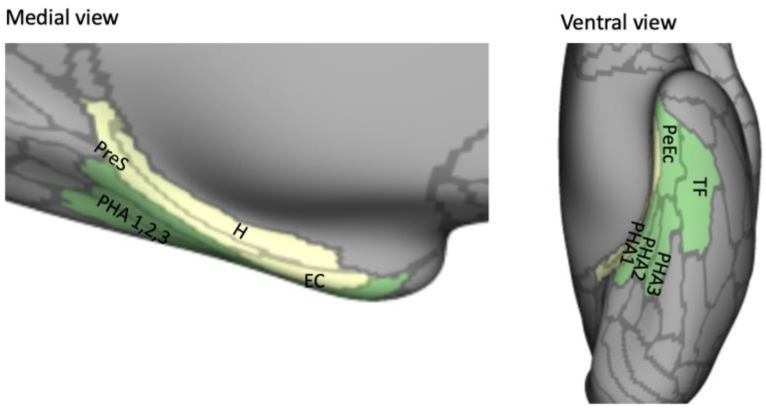

The hippocampal formation consists of the hippocampus, subicular complex, and entorhinal cortex (Insausti & Amaral, 2004). Additionally, the hippocampal voxels labeled by the Freesurfer automatic parcellation method implemented in fMRIprep (Esteban et al., 2019) were also included in the hippocampal formation. This added more voxels, especially in the superior regions, which were not originally included in the HCP MMP (Glasser et al., 2016).

We merged all remaining areas from the medial temporal lobe and referred to them as the parahippocampal region. This parahippocampal region included subregions such as the perirhinal-ectorhinal cortex, area TF, and parahippocampal areas (PHA1, PHA2, PHA3; Similar to Area TH (Ma et al., 2022)).

Supplementary Figure 4b. Brain regions and names used in the study

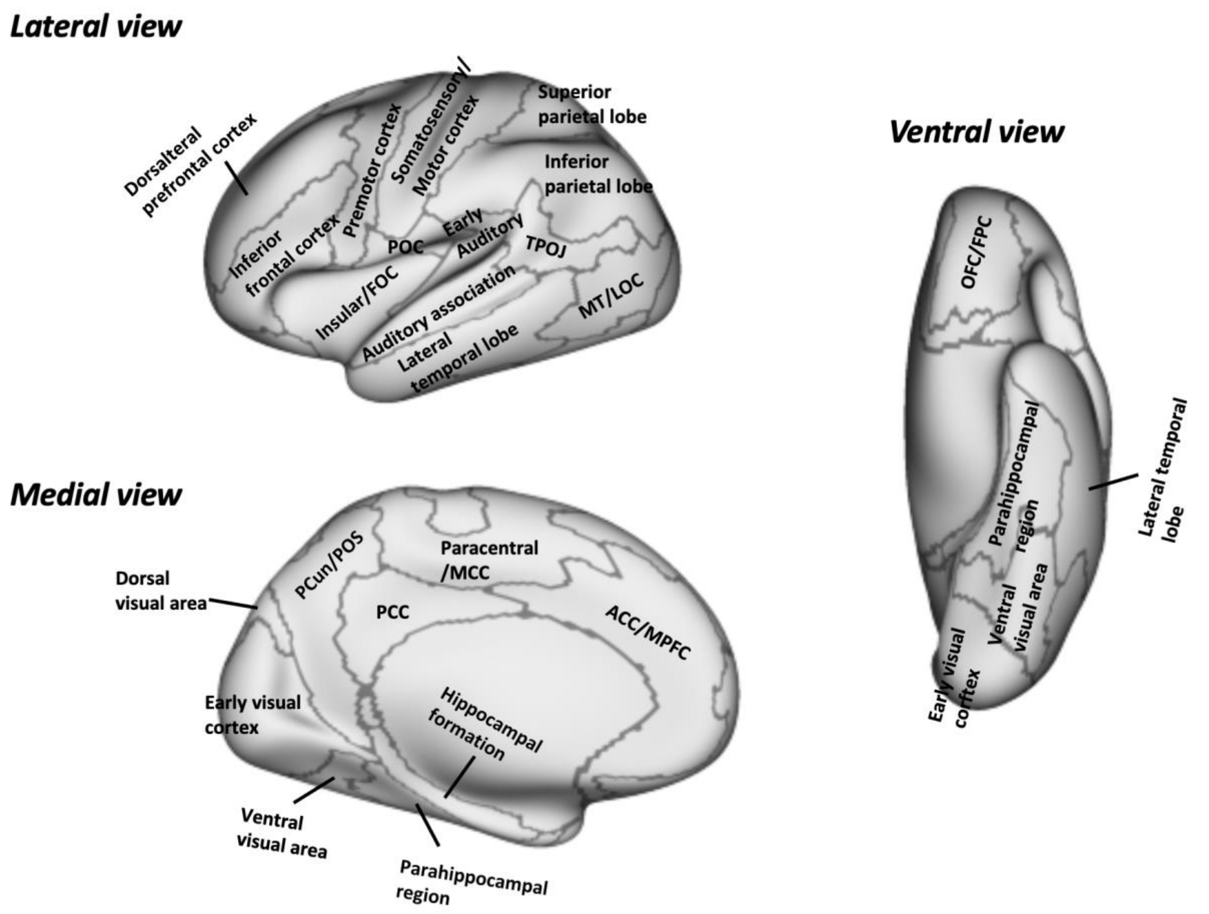

Abbreviations

TPOJ: Temporo-parieto-occipital junction; FOC: Frontal opercular cortex; POC: Posterior opercular cortex; MT: Middle temporal area; LOC: Lateral occipital cortex; PCun: Precuneus; POS: Parieto-occipital sulcus; Paracentral: Paracentral lobule; MCC: Middle cingulate cortex; ACC: Anterior cingulate cortex; MPFC: Medial prefrontal cortex; PCC: Posterior cingulate cortex; OFC: Orbitofrontal cortex; FPC: Frontopolar cortex

References (Supplementary Figure 2 ~ 4)

Baker, C. M., Burks, J. D., Briggs, R. G., Conner, A. K., Glenn, C. A., Manohar, K., Milton, C. K., Sali, G., McCoy, T. M., Battiste, J. D., O'Donoghue, D. L., & Sughrue, M. E. (2018). A Connectomic Atlas of the Human Cerebrum—Chapter 8: The Posterior Cingulate Cortex, Medial Parietal Lobe, and Parieto-Occipital Sulcus. *Operative Neurosurgery*, *15*(suppl_1), S350–S371. https://doi.org/10.1093/ons/opy262

Esteban, O., Markiewicz, C. J., Blair, R. W., Moodie, C. A., Isik, A. I., Erramuzpe, A., Kent, J. D., Goncalves, M., DuPre, E., Snyder, M., Oya, H., Ghosh, S. S., Wright, J., Durnez, J., Poldrack, R. A., & Gorgolewski, K. J. (2019). fMRIPrep: A robust preprocessing pipeline for functional MRI. *Nature Methods*, *16*(1), 111–116. https://doi.org/10.1038/s41592-018-0235-4

Glasser, M. F., Coalson, T. S., Robinson, E. C., Hacker, C. D., Harwell, J., Yacoub, E., Ugurbil, K., Andersson, J., Beckmann, C. F., Jenkinson, M., Smith, S. M., & Van Essen, D. C. (2016). A multi-modal parcellation of human cerebral cortex. *Nature*, *536*(7615), 171–178. https://doi.org/10.1038/nature18933

Huang, C.-C., Rolls, E. T., Feng, J., & Lin, C.-P. (2022). An extended Human Connectome Project multimodal parcellation atlas of the human cortex and subcortical areas. *Brain Structure and Function*, *227*(3), 763–778. https://doi.org/10.1007/s00429-021-02421-6

Insausti, R., & Amaral, D. G. (2004). Hippocampal formation. *The Human Nervous System*, *2*, 871–914.

Ma, Q., Rolls, E. T., Huang, C.-C., Cheng, W., & Feng, J. (2022). Extensive cortical functional connectivity of the human hippocampal memory system. *Cortex*, *147*, 83–101. https://doi.org/10.1016/j.cortex.2021.11.014

Nasr, S., Liu, N., Devaney, K. J., Yue, X., Rajimehr, R., Ungerleider, L. G., & Tootell, R. B. H. (2011). Scene-Selective Cortical Regions in Human and Nonhuman Primates. *The Journal of Neuroscience*, *31*(39), 13771–13785. https://doi.org/10.1523/JNEUROSCI.2792-11.2011

Rolls, E. T., Wirth, S., Deco, G., Huang, C., & Feng, J. (2023). The human posterior cingulate, retrosplenial, and medial parietal cortex effective connectome, and implications for memory and navigation. *Human Brain Mapping*, *44*(2), 629–655. https://doi.org/10.1002/hbm.26089

Silson, E. H., Steel, A. D., & Baker, C. I. (2016). Scene-Selectivity and Retinotopy in Medial Parietal Cortex. *Frontiers in Human Neuroscience*, *10*. https://doi.org/10.3389/fnhum.2016.00412

Yamaguchi, A., & Jitsuishi, T. (2023). Structural connectivity of the precuneus and its relation to resting-state networks. *Neuroscience Research*, S0168010223002213. https://doi.org/10.1016/j.neures.2023.12.004

Supplementary Table 1. Statistical test results for mediation analyses to characterize the role of ICS or intersubject functional connectivity in age-related spatial memory decline.

Total effect: $Y = cX + d_1Z + i_1 + e_1$

Mediation: $M = aX + d_2Z + i_1 + e_2$

$$Y = c'X + bM + d_3Z + i_3 + e_3$$

The prerequisite of mediation analysis is that the mediator variable (M) must have a relationship with the independent variable (X), so ICS or intersubject functional connectivity (FC) with the hippocampal formation significantly correlated with age was considered for further mediation analyses. All coefficients and statistical significance from the mediation analyses are listed in the table below:

ICS

Region	Hemisphere	β_a	β_b	$\beta_{c'}$	p_a	p_b	$p_{c'}$	β_{a*b} (Mediation effect)	Sobel *z*	p
Parahippocampal region	**(L)**	**-0.318**	**0.313**	**-0.597**	**0.016**	**< 0.001**	**< 0.001**	**-0.099**	**-2.096**	**0.036**
	(R)	-0.275	0.222	-0.636	0.034	0.010	< 0.001	-0.061	-1.668	0.095
Ventral visual area	(L)	-0.284	0.086	-0.673	0.022	0.356	< 0.001	-0.024	-0.864	0.388
Early visual cortex	(L)	-0.271	0.122	-0.664	0.027	0.193	< 0.001	-0.033	-1.136	0.256
Lateral temporal lobe	(R)	-0.265	0.117	-0.666	0.043	0.180	< 0.001	-0.031	-1.132	0.258
Inferior frontal cortex	(L)	-0.262	0.060	-0.681	0.049	0.486	< 0.001	-0.016	-0.661	0.508
Posterior opercular cortex	(R)	0.315	0.107	-0.731	0.017	0.217	< 0.001	0.034	1.108	0.268

Intersubject FC with the hippocampal formation

Region	Hemisphere	β_a	β_b	$\beta_{c'}$	p_a	p_b	$p_{c'}$	β_{a*b} (Mediation effect)	Sobel z	p
Superior parietal lobe	(L)	-0.467	0.168	-0.618	< 0.001	0.076	< 0.001	-0.079	-1.638	0.101
	(R)	-0.488	0.202	-0.598	< 0.001	0.029	< 0.001	-0.099	-1.951	0.051
Premotor cortex	**(L)**	**-0.469**	**0.226**	**-0.591**	**< 0.001**	**0.013**	**< 0.001**	**-0.106**	**-2.130**	**0.033**
	(R)	-0.420	0.176	-0.623	0.001	0.048	< 0.001	-0.074	-1.725	0.085
Dorsal visual area	(L)	-0.376	0.174	-0.631	0.003	0.059	< 0.001	-0.066	-1.634	0.102
	(R)	-0.461	0.188	-0.610	< 0.001	0.038	< 0.001	-0.087	-1.843	0.065
Parahippocampal region	**(L)**	**-0.413**	**0.292**	**-0.576**	**0.001**	**< 0.001**	**< 0.001**	**-0.120**	**-2.404**	**0.016**
MT/LOC	**(L)**	**-0.376**	**0.229**	**-0.611**	**0.003**	**0.011**	**< 0.001**	**-0.086**	**-1.984**	**0.047**
	**(R)**	**-0.405**	**0.227**	**-0.605**	**0.002**	**0.011**	**< 0.001**	**-0.092**	**-2.040**	**0.041**
Ventral visual area	(L)	-0.287	0.276	-0.618	0.026	0.001	< 0.001	-0.079	-1.876	0.061
	**(R)**	**-0.371**	**0.225**	**-0.613**	**0.005**	**0.010**	**< 0.001**	**-0.083**	**-1.966**	**0.049**
Paracentral/MCC	(L)	-0.343	0.201	-0.628	0.009	0.020	< 0.001	-0.069	-1.778	0.075
	(R)	-0.362	0.202	-0.624	0.006	0.020	< 0.001	-0.073	-1.821	0.069
PCun/POS	**(L)**	**-0.343**	**0.273**	**-0.603**	**0.008**	**0.002**	**< 0.001**	**-0.094**	**-2.094**	**0.036**
	**(R)**	**-0.349**	**0.244**	**-0.612**	**0.008**	**0.004**	**< 0.001**	**-0.085**	**-1.998**	**0.046**

Regions showing statistically significant mediation effects ($p < 0.05$) are indicated in bold.

Supplementary Table 2. Reanalysis of aging effects on ICS and intersubject FC using age as a categorical variable.

The age distribution of participants in this experiment was bimodal, suggesting that estimating group differences in ICS (i.e., comparing young vs. older participants) between the two age groups may also be valid. Within the regression framework, this can be implemented by using a categorical age variable (young = 0, aging = 1) as a predictor instead of the z-scored age.

ICS (or ISFC) = a × age_cate + b × motion + c

In the mediation analysis, the categorical age variable (coded as 0 for young and 1 for aging) was also z-scored to obtain standardized coefficients in the regression models.

Aging effect on ICS

Region	hemi	*Age regression model* Coefficient	p-value	*Mediation effects* Indirect effect	Sobel z	p-value
Parahippocampal region	(L)	**-0.6163**	**0.0218**	**-0.0980**	**-2.0263**	**0.0427**
	(R)	**-0.5302**	**0.0457**	-0.0607	-1.6312	0.1029
Ventral visual area	(L)	**-0.5493**	**0.0306**	-0.0267	-0.9562	0.3390
Early visual cortex	(L)	**-0.5046**	**0.0440**	-0.0347	-1.2081	0.2270
Posterior opercular cortex	(R)	**0.7162**	**0.0077**	0.0442	1.2637	0.2064

Aging effect on intersubject FC with the hippocampal formation

Region	hemi	*Age regression model* Coefficient	p-value	*Mediation effects* Indirect effect	Sobel z	p-value
Superior parietal lobe	(L)	**-0.8578**	**0.0008***	-0.0840	-1.8364	0.0663
	(R)	**-0.8621**	**0.0010***	**-0.1011**	**-2.1184**	**0.0341**
Premotor cortex	(L)	**-0.8364**	**0.0016***	**-0.1061**	**-2.2075**	**0.0273**
	(R)	**-0.7402**	**0.0058**	-0.0757	-1.8391	0.0659
Dorsal visual area	(L)	**-0.6167**	**0.0171**	-0.0658	-1.7262	0.0843

	(R)	**-0.8121**	**0.0022***	**-0.0892**	**-1.9914**	**0.0464**
Parahippocampal region	(L)	**-0.7142**	**0.0076**	**-0.1130**	**-2.2561**	**0.0241**
MT/LOC	(L)	**-0.5743**	**0.0283**	-0.0783	-1.8455	0.0650
	(R)	**-0.6946**	**0.0087**	**-0.0891**	**-2.0229**	**0.0431**
Paracentral/MCC	(L)	**-0.6309**	**0.0191**	-0.0686	-1.7651	0.0776
	(R)	**-0.6744**	**0.0123**	-0.0732	-1.8259	0.0679
PCun/POS	(L)	**-0.6243**	**0.0182**	**-0.0901**	**-1.9912**	**0.0465**
	(R)	**-0.6507**	**0.0159**	-0.0837	-1.9429	0.0520
Ventral visual area	(R)	**-0.6099**	**0.0237**	-0.0783	-1.8624	0.0625

Bold font indicates statistical significance ($p < 0.05$). Asterisks indicate statistical significance after multiple comparison correction (FDR adjusted $p < 0.05$).

Supplementary Figure 5-7. Across map differences in ICS and decoding accuracy

We examined differences in ICS across navigation episodes (maps) to provide insights into which characteristics of a navigation episode induced shared brain activities across participants. Since our previous analysis showed that turn- and landmark-evoked activity contributed to the canonical dynamics in many brain regions, we characterized each navigation episode by (i) the presence or absence of local landmarks and (ii) the total change in orientation. For each region and each navigation episode, the mean ICS was calculated across all participants.

Supplementary Figure 5. A higher ICS in episodes with landmarks

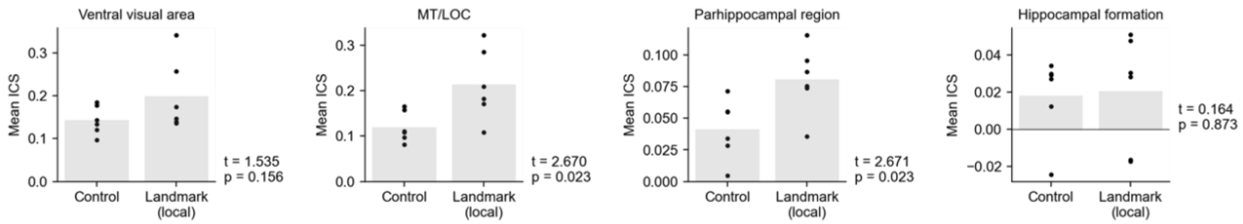

First, we compared the ICS between no landmarks (control) and landmark conditions in the brain regions where canonical dynamics were influenced by landmark-evoked activation (i.e. early visual cortex, ventral visual area, MT/LOC, parahippocampal region). As expected, we found significant differences in some of the regions (MT/LOC: $t(10) = 2.670$, $p = 0.023$; parahippocampal region: $t(10) = 2.671$, $p = 0.023$) and a trend in the ventral visual area ($t(10) = 1.535$, $p = 0.156$). However, there were no significant differences in the hippocampal ICS between episodes with and without landmarks ($t(10) = 0.164$, $p = 0.873$).

Supplementary Figure 6. Improved decoding accuracy in episodes with landmarks

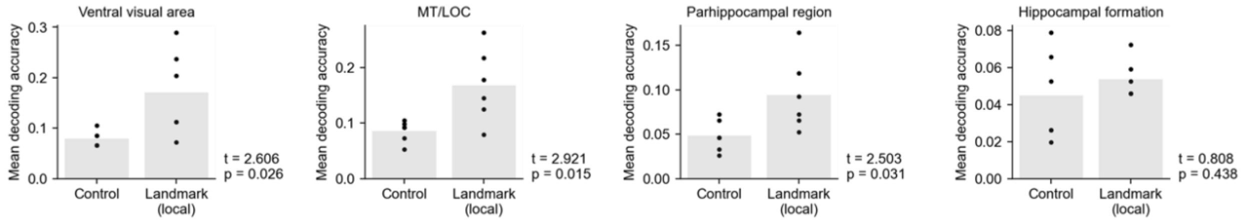

Similarly, we compared the decoding accuracy between episodes with and without landmarks for each brain region. In line with the ICS results, the ventral visual area ($t(10)=2.606$, $p=0.026$),

MT/LOC (t(10)=2.921, p=0.015), and parahippocampal region (t(10) =2.503, p=0.031) showed higher decoding accuracy in episodes with local landmarks. Again, the hippocampal formation did not show a difference (t(10) =1.07, p=0.29). These results suggested that landmarks during navigation tend to make participants' brain dynamics more synchronized and more distinguishable from other episodes.

Supplementary Figure 7. A higher ICS with a higher degree of turning

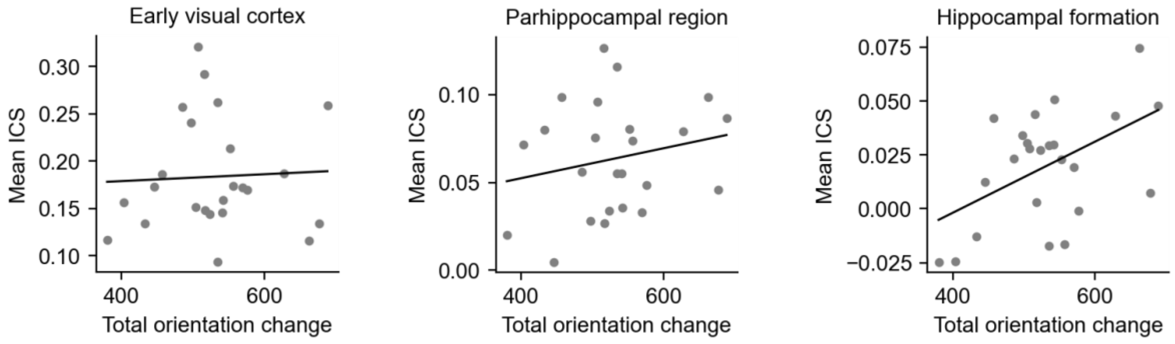

To characterize a relationship between ICS and a degree of turning, we defined a total orientation change by summing all absolute path angle changes during the whole period of one navigational episode. Each dot represents one episode, and for simplicity, we averaged the ICS from both hemispheres. There was no significant relationship between mean ICS and total orientation change in the early visual cortex (r = 0.048, p = 0.82) and the parahippocampal region (r = 0.213, p = 0.327), which significantly showed turn-evoked signals in its canonical dynamics, showed an insignificant trend of a positive correlation. However, in the hippocampal formation, ICS differences across episodes were significantly correlated with total orientation change (r = 0.502, p = 0.012), even though its canonical dynamics did not significantly reflect turn-evoked activities.

We recognize that these results may not be entirely conclusive, as each episode varies across multiple dimensions, and this analysis is based on a limited number of episodes (n = 24), in contrast to our primary analysis on individual differences (n = 76). Future studies could further clarify these across-episode ICS differences, investigating contributors to ICS and addressing the discrepancy between (i) the relationship between canonical dynamics and turn-evoked activity, and (ii) the relationship between mean ICS and total orientation change across episodes, as we have described here.

Supplementary Figure 8 and Table 3. Individual differences in ICS of hippocampal formation and its intersubject functional connectivity with other regions

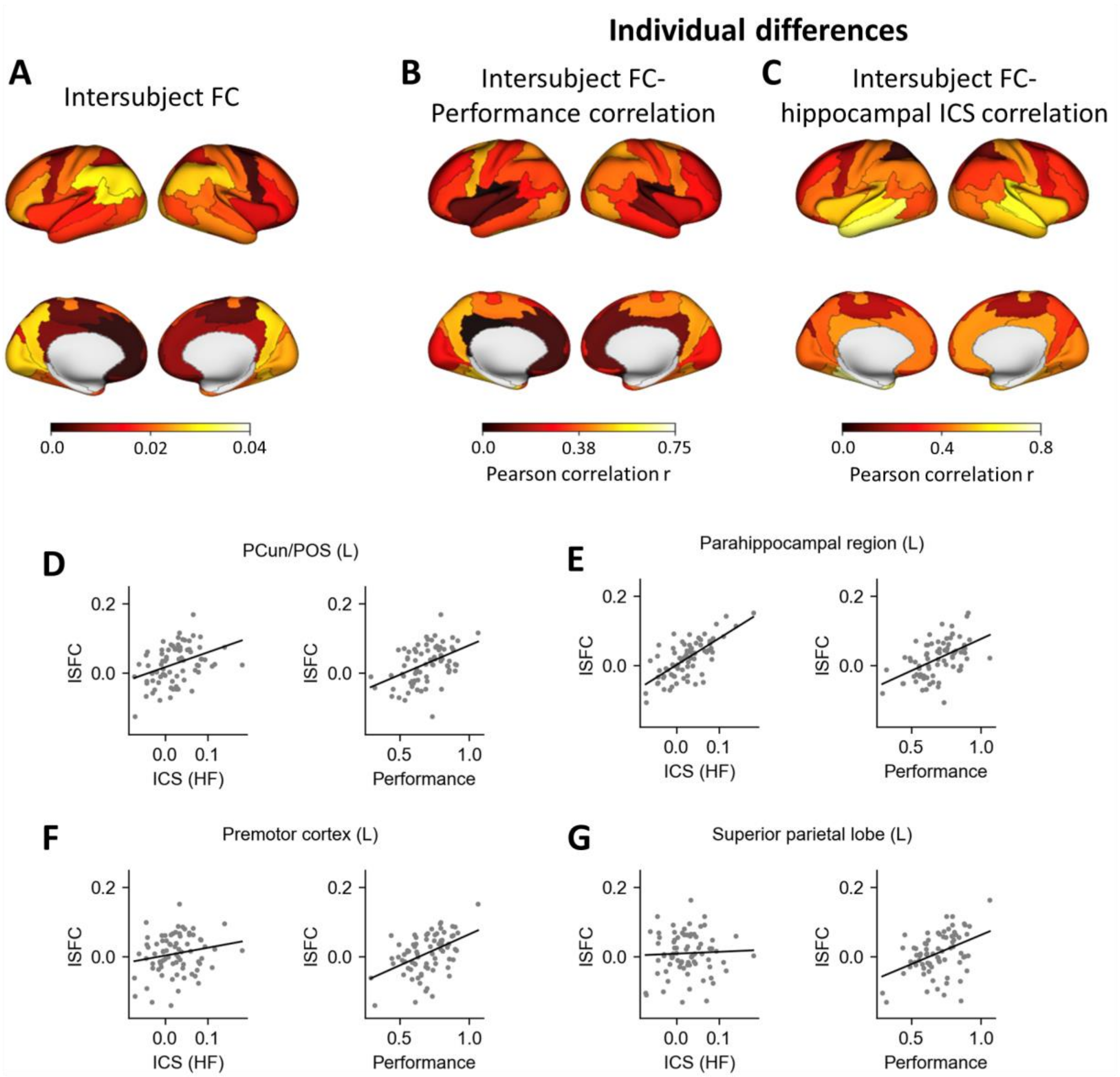

(A) The hippocampal intersubject functional connectivity (FC) was measured by averaging a correlation between individual hippocampal dynamics and canonical cortical dynamics across all participants. **(B)** The intersubject FC in many regions was strongly correlated with performance. **(C)** In addition, the hippocampal ICS was correlated with the intersubject FC in wide brain regions (see the table below). In brain regions with higher intersubject FC, such as the PCun/POS **(D)** and parahippocampal region **(E)**, their intersubject FC was highly correlated with both hippocampal ICS and performance. Moreover, the superior parietal lobe **(F)** and premotor cortex

(G) also showed a strong correlation between their intersubject FC and memory performance despite their lower intersubject FC and a weak correlation with the hippocampal ICS. These results implied that broader cortical regions are functionally coupled to the hippocampal formation in varying degrees and contribute to better memory performance.

		Intersubject FC with the hippocampal formation		A correlation between performance and intersubject FC		A correlation between ICS and intersubject FC	
Region	Hemisphere	Mean	Standard error	r	p	r	p
Inferior parietal lobe	(L)	0.0281	0.0061	0.3357	0.0030	0.4287	0.0001
	(R)	0.0260	0.0064	0.3832	0.0006	0.3358	0.0030
PCun/POS	(L)	0.0266	0.0064	0.4573	< 0.0001	0.3793	0.0007
	(R)	0.0270	0.0070	0.4345	0.0001	0.3416	0.0025
Early visual cortex	(L)	0.0264	0.0061	0.2417	0.0354	0.3940	0.0004
	(R)	0.0238	0.0061	0.2818	0.0137	0.4334	0.0001
TPOJ	(L)	0.0284	0.0074	0.2751	0.0162	0.3851	0.0006
	(R)	0.0201	0.0068	0.3104	0.0063	0.3929	0.0004
Ventral visual area	(L)	0.0213	0.0065	0.4348	0.0001	0.4515	< 0.0001
	(R)	0.0262	0.0065	0.4252	0.0001	0.4401	0.0001
Somatosensory/Motor cortex	(L)	0.0208	0.0064	0.2934	0.0101	0.4237	0.0001
	(R)	0.0204	0.0063	0.3091	0.0066	0.4644	< 0.0001
Parahippocampal region	(L)	0.0192	0.0061	0.5050	< 0.0001	0.6975	< 0.0001
	(R)	0.0197	0.0058	0.3745	0.0009	0.4947	< 0.0001
Lateral temporal lobe	(L)	0.0166	0.0059	0.3253	0.0041	0.6567	< 0.0001
	(R)	0.0218	0.0059	0.2850	0.0126	0.5258	< 0.0001
Posterior opercular cortex	(L)	0.0181	0.0060	-0.0209	0.8579	0.4184	0.0002
	(R)	0.0200	0.0061	0.0319	0.7846	0.5047	< 0.0001
Inferior frontal cortex	(L)	0.0240	0.0056	0.3107	0.0063	0.4004	0.0003
	(R)	0.0136	0.0072	0.2704	0.0182	0.3360	0.0030
Dorsolateral prefrontal	(L)	0.0195	0.0070	0.2431	0.0343	0.3058	0.0072
	(R)	0.0178	0.0087	0.2142	0.0632	0.3248	0.0042
Early auditory cortex	(L)	0.0158	0.0060	0.0747	0.5215	0.5158	< 0.0001
	(R)	0.0208	0.0063	0.0725	0.5334	0.5941	< 0.0001
MT/LOC	(L)	0.0175	0.0066	0.4293	0.0001	0.3631	0.0013

	(R)	0.0179	0.0056	0.4410	0.0001	0.4151	0.0002
Auditory association area	(L)	0.0153	0.0067	0.0753	0.5178	0.5624	< 0.0001
	(R)	0.0183	0.0061	0.1074	0.3560	0.6159	< 0.0001
Dorsal visual area	(L)	0.0116	0.0063	0.3798	0.0007	0.1443	0.2137
	(R)	0.0197	0.0064	0.4299	0.0001	0.2572	0.0249
Insular/FOC	(L)	0.0163	0.0069	0.0825	0.4789	0.5101	< 0.0001
	(R)	0.0112	0.0067	0.2085	0.0707	0.5268	< 0.0001
Superior parietal lobe	(L)	0.0097	0.0070	0.4166	0.0002	0.0436	0.7083
	(R)	0.0110	0.0069	0.4533	< 0.0001	0.1138	0.3276
PCC	(L)	0.0058	0.0063	-0.0308	0.7920	0.4452	0.0001
	(R)	0.0088	0.0067	0.0986	0.3968	0.4517	< 0.0001
OFC/FPC	(L)	0.0058	0.0058	0.1706	0.1405	0.3197	0.0049
	(R)	0.0058	0.0060	0.2854	0.0125	0.3644	0.0012
Premotor cortex	(L)	0.0082	0.0065	0.4667	< 0.0001	0.1923	0.0961
	(R)	0.0033	0.0067	0.4007	0.0003	0.2530	0.0275
ACC/MPFC	(L)	0.0015	0.0066	0.0473	0.6847	0.4142	0.0002
	(R)	0.0073	0.0075	0.0837	0.4721	0.4443	0.0001
Paracentral/MCC	(L)	0.0045	0.0063	0.3900	0.0005	0.1856	0.1085
	(R)	0.0040	0.0065	0.3992	0.0004	0.1608	0.1652

Supplementary Figure 9. Relationship between memory performance and intersubject functional connectivity using cortical regions as seed regions.

Seed regions in intersubject functional connectivity refer to regions whose individual dynamics are correlated with the canonical dynamics of other brain regions. In the main analysis, we used the hippocampal formation as the seed region (see Figure 5A). We conducted a similar analysis using other brain regions as seed regions. In this analysis, brain regions were defined bilaterally to reduce complexity.

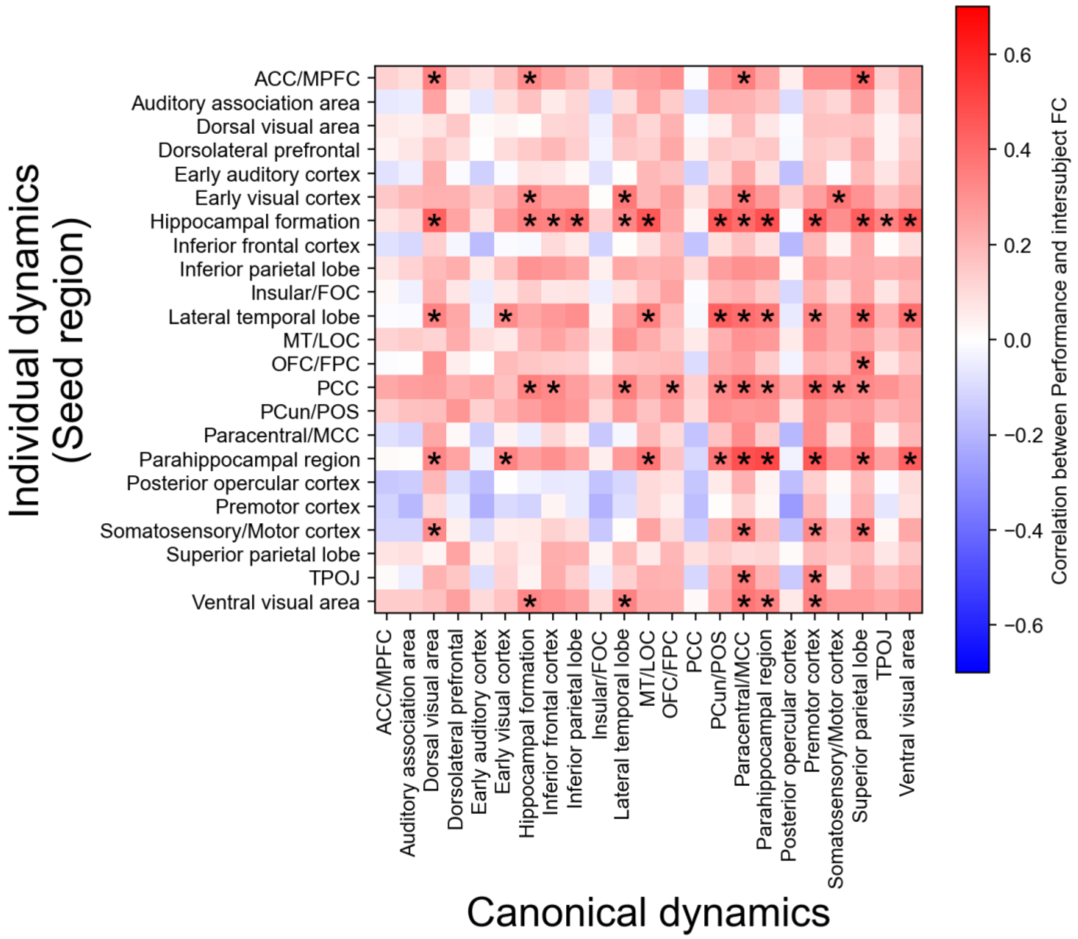

Heat map plot visualizes the correlation between memory performance and intersubject FC (see color bar). Asterisks indicate statistically significant values (*: FDR-adjusted $p < 0.05$).

The relationship between memory performance and intersubject functional connectivity (FC) with overall cortical regions (averaged across columns in the heatmap above) was visualized

using the horizontal bar plot below. The order of regions was rearranged in descending order based on their mean performance relevance. The performance relevance of intersubject FC was strongest when the hippocampal formation was used as the seed region (mean r = 0.296), which may be due to the navigational nature of our task.

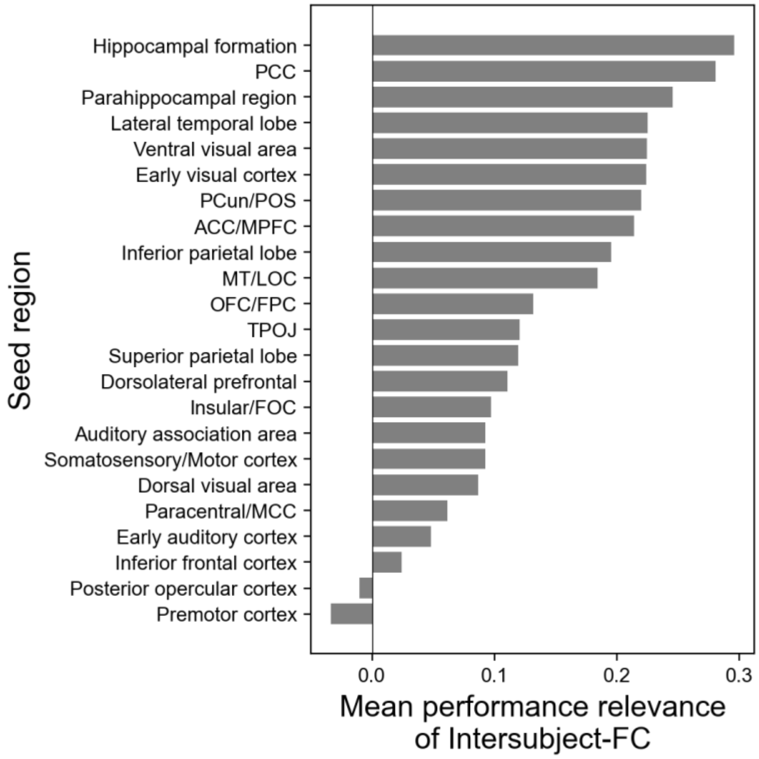

Supplementary Figure 10. Difference in overall ICS when using canonical dynamics derived from only young participants, only aging participants, or all participants.

We calculated ICS using three different canonical dynamics: (i) average dynamics from only younger participants, (ii) average dynamics from only aging participants, and (iii) average dynamics from all participants.

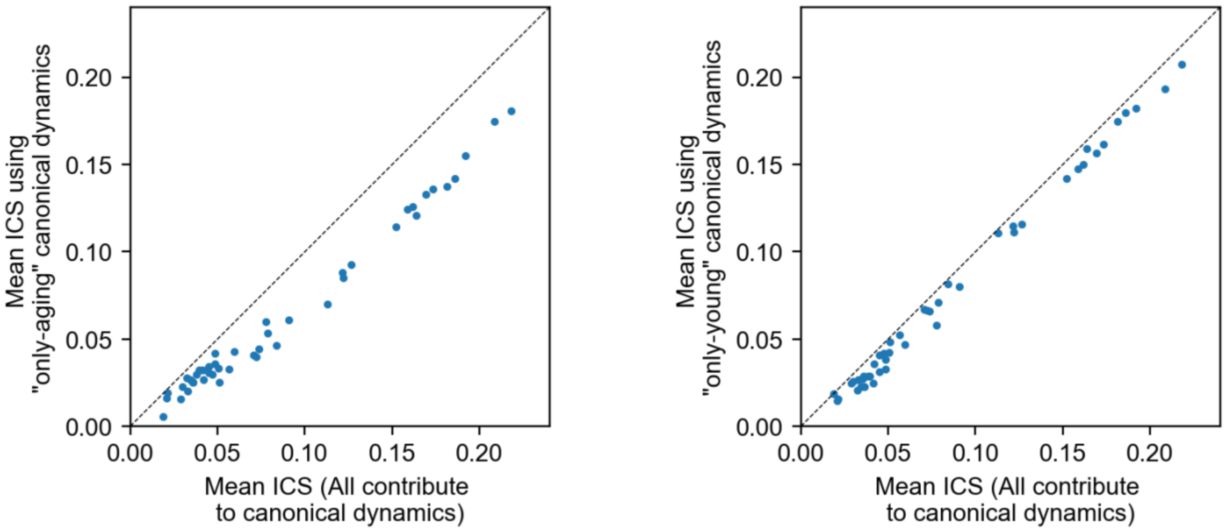

The plot shows the mean ICS across all participants for each method. Each dot represents a brain region, and the dashed line indicates the identity line. ICS values computed using older participants' average dynamics as the canonical model were lower than those computed using all participants' data ($t = 13.52$, $p < 0.001$). Similarly, ICS values using only younger participants' data were also lower than those using all participants ($t = 11.73$, $p < 0.001$). These results confirm that incorporating both age groups provides the most reliable canonical dynamics.

Supplementary Figure 11. Details of analysis on eye-tracking data.

Eye-tracking data were collected from an independent group of participants recruited for a prior EEG experiment with eye-tracking, using the same paradigm and stimuli as in the fMRI study described in the main manuscript (See Methods).

Gaze activity was recorded using a Tobii Pro Fusion eye tracker at a sampling rate of 250 Hz. Nineteen (4 were under age 40 and 15 were over age 40) out of 61 participants were excluded from the gaze analysis due to invalid data caused by head movements or light reflections from glasses.

Objects in the video were manually annotated in each frame as either a local landmark (referred to as "landmark" in the main text), a distal cue, or an uninformative building. Gaze indices for each participant were computed frame-by-frame based on the spatial overlap between the annotated "local landmark" regions and a 100-pixel diameter circle centered on the participant's gaze coordinates. For further analysis, the gaze index was resampled to 10 Hz.

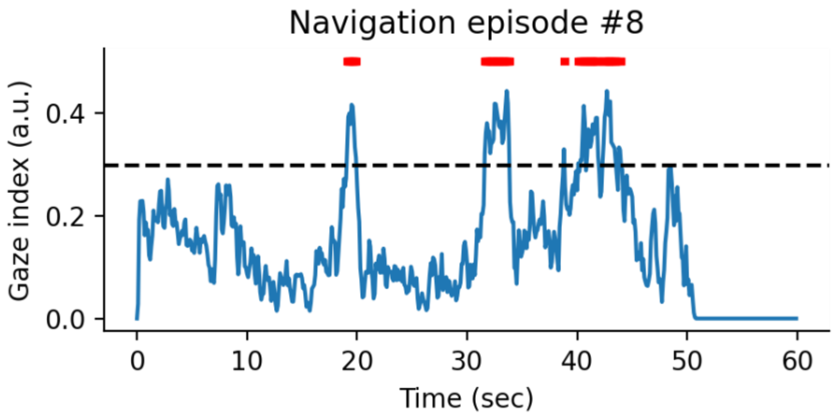

To determine when people viewed the landmark (see Fig. 3 in the main text), we averaged the gaze index across all valid participants at each time point (blue line in the plot).

To define the criterion for landmark viewing, we concatenated the averaged gaze index across all time points from all navigation episodes (600 time points × 24 episodes). We then calculated the 10th percentile of this distribution (0.2985; indicated by the dashed line in the plot) as the

threshold for landmark viewing. The time points at which the average gaze index exceeded this threshold were defined as the landmark viewing period (highlighted in red in the plot).

To verify that there was no significant difference in landmark viewing or task engagement based on participants' age, we divided participants into two groups (young: under 40; aging: over 40) and compared their gaze indices. The age of the participants after exclusion ranged from 22 to 63 years (n = 42, 24 females; M = 40.48, SD = 13.37). When the participants were divided into two groups based on age (under or over 40), the young group ranged from 22 to 38 years (n = 21, 10 females; M = 27.95, SD = 3.99), and the aging group ranged from 41 to 63 years (n = 21, 14 females; M = 53.00, SD = 5.30). Although the age variability (i.e., SD) within both groups was slightly greater than in the fMRI experiment (see Methods), the lack of difference in gaze behavior between young and aging participants in the eye-tracking data suggests a similar pattern in the fMRI participants as well.

The gaze index did not significantly differ between the young group (mean = 0.098, SD = 0.030; n = 21) and the aging group (mean = 0.091, SD = 0.035; n = 21), ($t(40) = 0.675$, $p = 0.503$). This result suggests that both young and aging participants focused similarly on the landmarks during the navigation episodes and were equally well engaged in the task.
